# Supplementary material for: Humans display interindividual differences in the latent mechanisms underlying fear generalization behaviour
Source: Commun Psychol. 2023 Aug 1;1:5. doi: 10.1038/s44271-023-00005-0 (PMC11290606; doi:10.1038/s44271-023-00005-0)
Supplement: Supplementary file 1 — Supplementary Information [file 44271_2023_5_MOESM1_ESM.pdf]

Supplementary Information: Humans display interindividual differences in the latent mechanisms underlying fear generalization behaviour

Kenny Yu[1,*], **Francis Tuerlinckx**[1], **Wolf Vanpaemel**[1], **and Jonas Zaman**[1,2]

[1]KU Leuven, Leuven, Belgium
[2]University of Hasselt, Hasselt, Belgium
[*]kenny.yu@kuleuven.be

This document contains additional modelling information and results to the manuscript: **Humans display interindividual differences in the latent mechanisms underlying fear generalization behaviour**. The raw data and the codes for analysis can be accessed from https://osf.io/sxjak/. Contact Kenny Yu (kenny.yu@kuleuven.be) for more information or questions about the manuscript.

Supplementary Note 1

In this manuscript, we define *Overgeneralizers* as those participants who generalize less than 70% of their learning even when encountering the most physically distant stimulus. In Figure 1, Panel A demonstrates that when the most distant distance measures 68.62 mm in circle diameter, learning decreases by more than 70% when the λ value exceeds .0052. Furthermore, Panel B of Figure 1 illustrates the stimuli similarity between stimuli, where λ is set to .0052, ranging from a distance of 0 to 100. Note that the specified boundary for λ is sensitive to variations in the range of numerical values considered. It is important to acknowledge that different ranges may yield different thresholds for the decrease in learning exceeding 70% when encountering the most physically distant stimulus. Therefore, researchers should be mindful of this sensitivity when interpreting the results and considering alternative ranges for λ in their investigations.

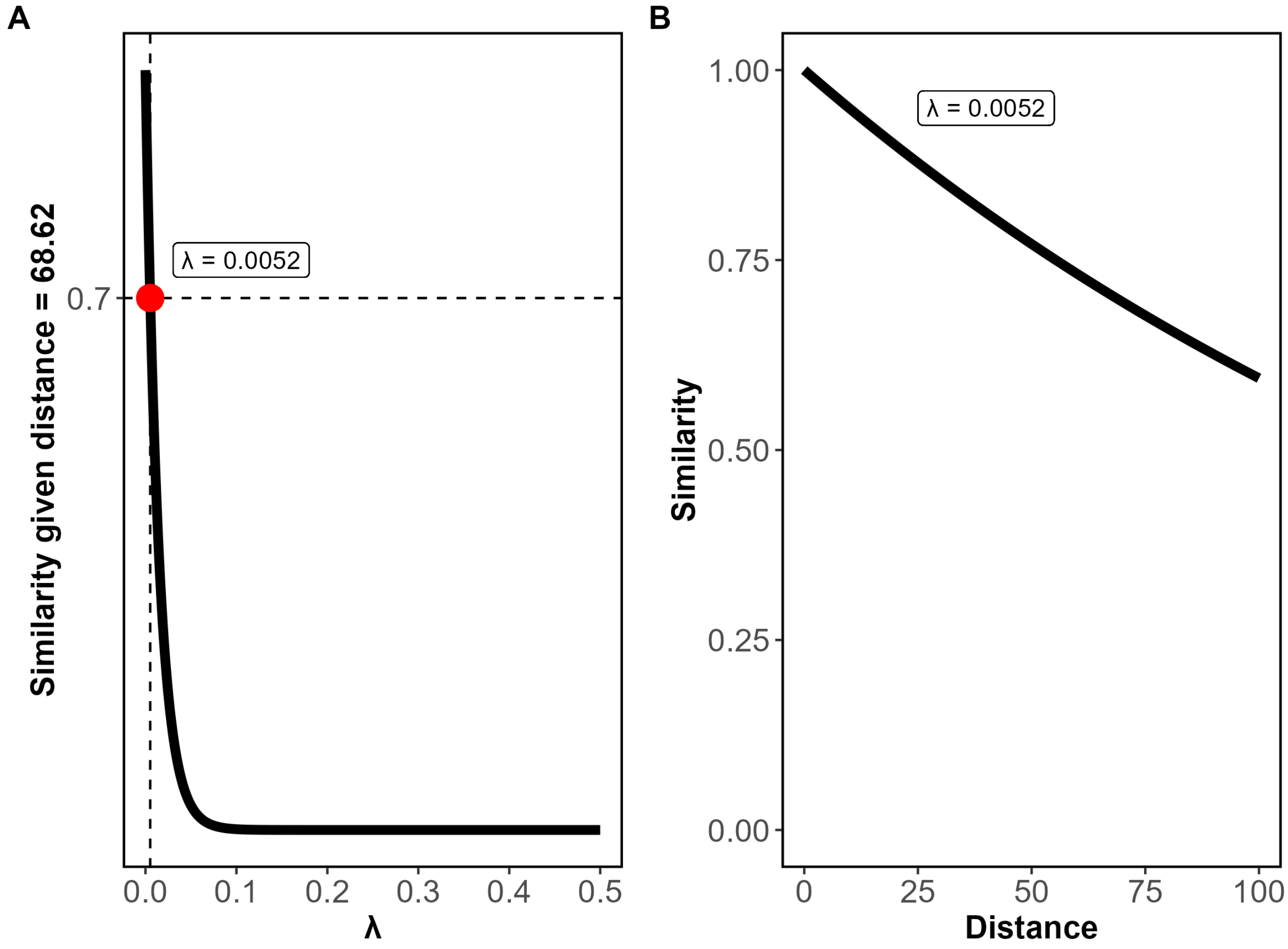

Supplementary Figure 1. Panel A: The similarity as a function of a changing λ for a particular distance: a physically very different stimulus (distance = 68.62). Panel B: The response decay curve with the upper boundary value for the generalization rate λ of *Overgeneralizers*

.

To check how sensitive our results are to the λ_i boundary value of .0052. We check how many posterior samples of λ_i are cling to .0052. It is shown in Figure 2 that most of λ posterior samples are not close to the boundary value of .0052.

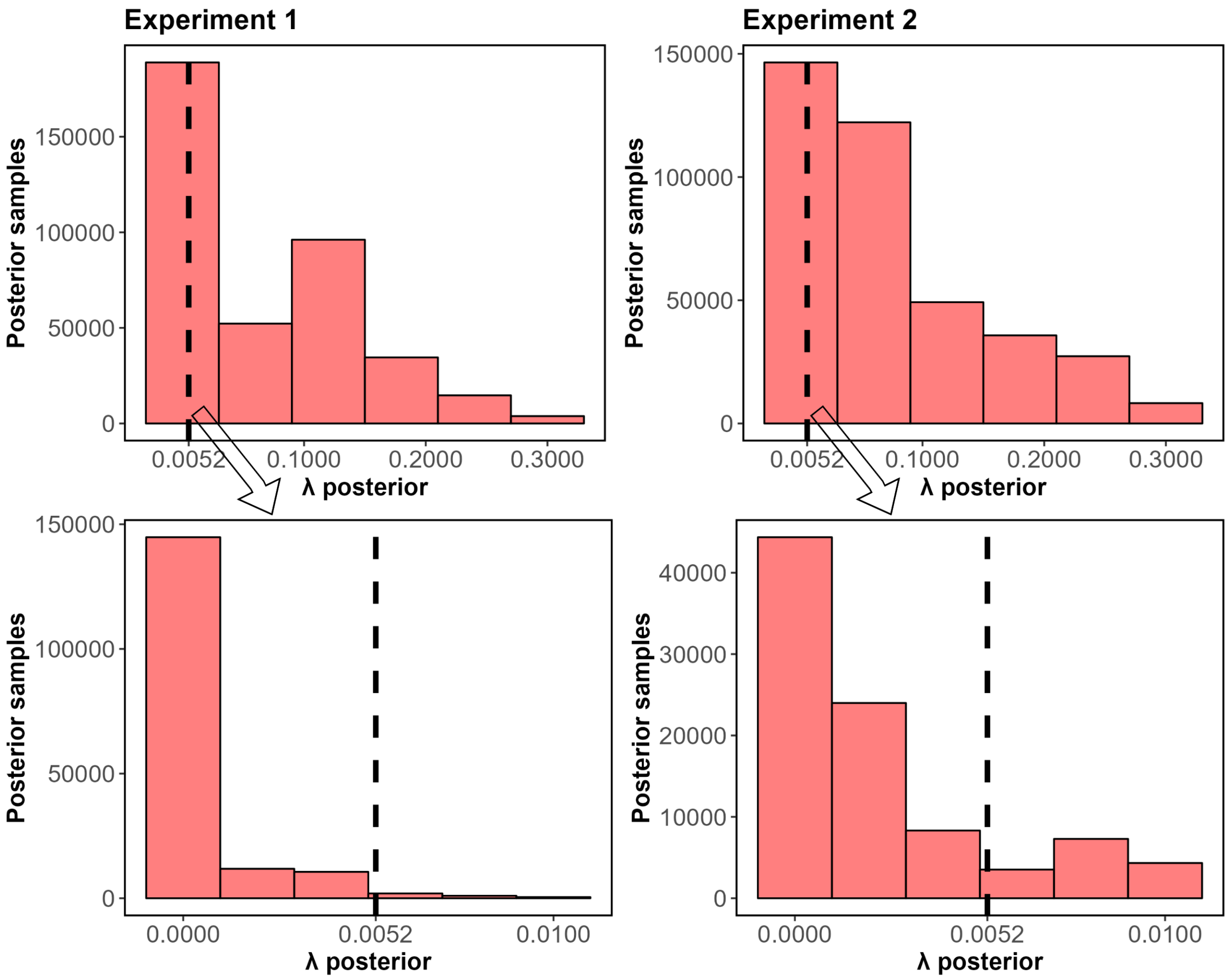

Supplementary Figure 2. Histogram of $lambda_i$ poterior samples of both Experiment 1 and 2.

.

Supplementary Note 2

In Figure 3 we show how different values of scaling parameters w0 and w1 will lead to different transformation between the latent response g and observed response θ.

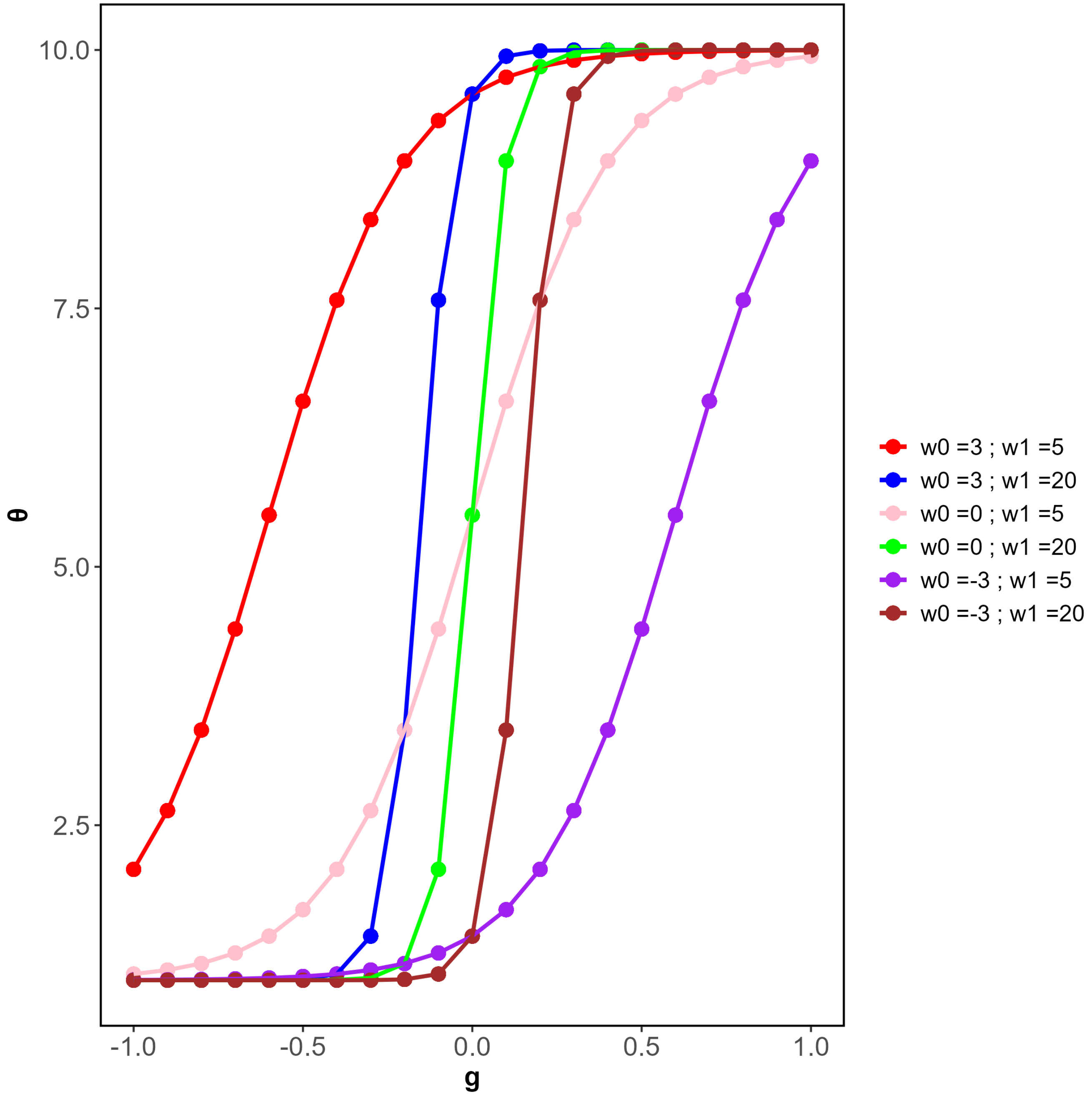

Supplementary Figure 3. The transformations between the latent response g and observed response θ with different combinations of the base line response parameter $w0$ and scaling parameter $w1$.

Supplementary Note 3

From Figure 4 to Figure 8, we show results of group allocation based on different proportion criteria of the group allocation parameter posterior being sampled to the same value.

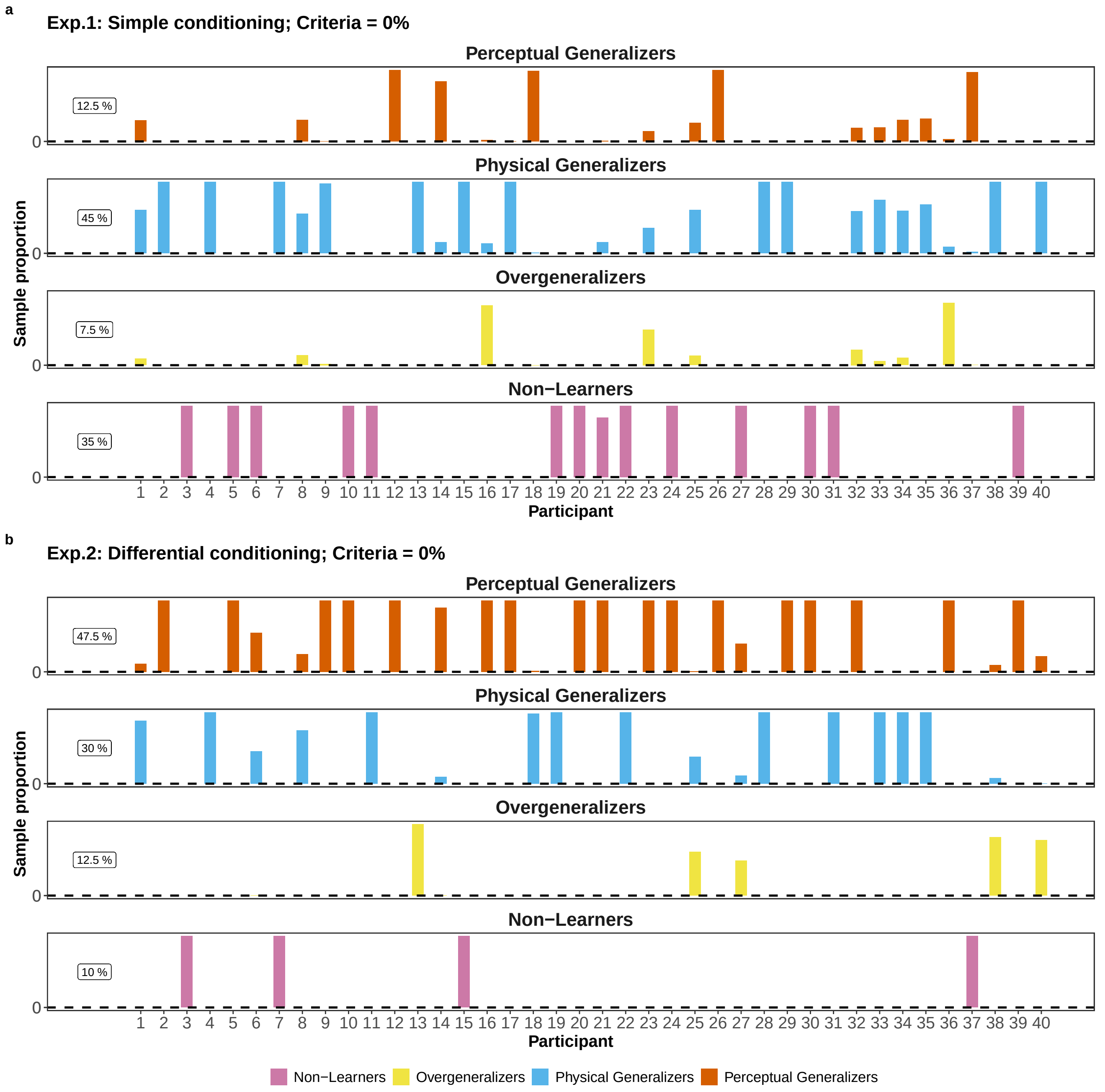

Supplementary Figure 4. The results of group allocation based on the criteria of the most frequent sampling value of the membership parameter m

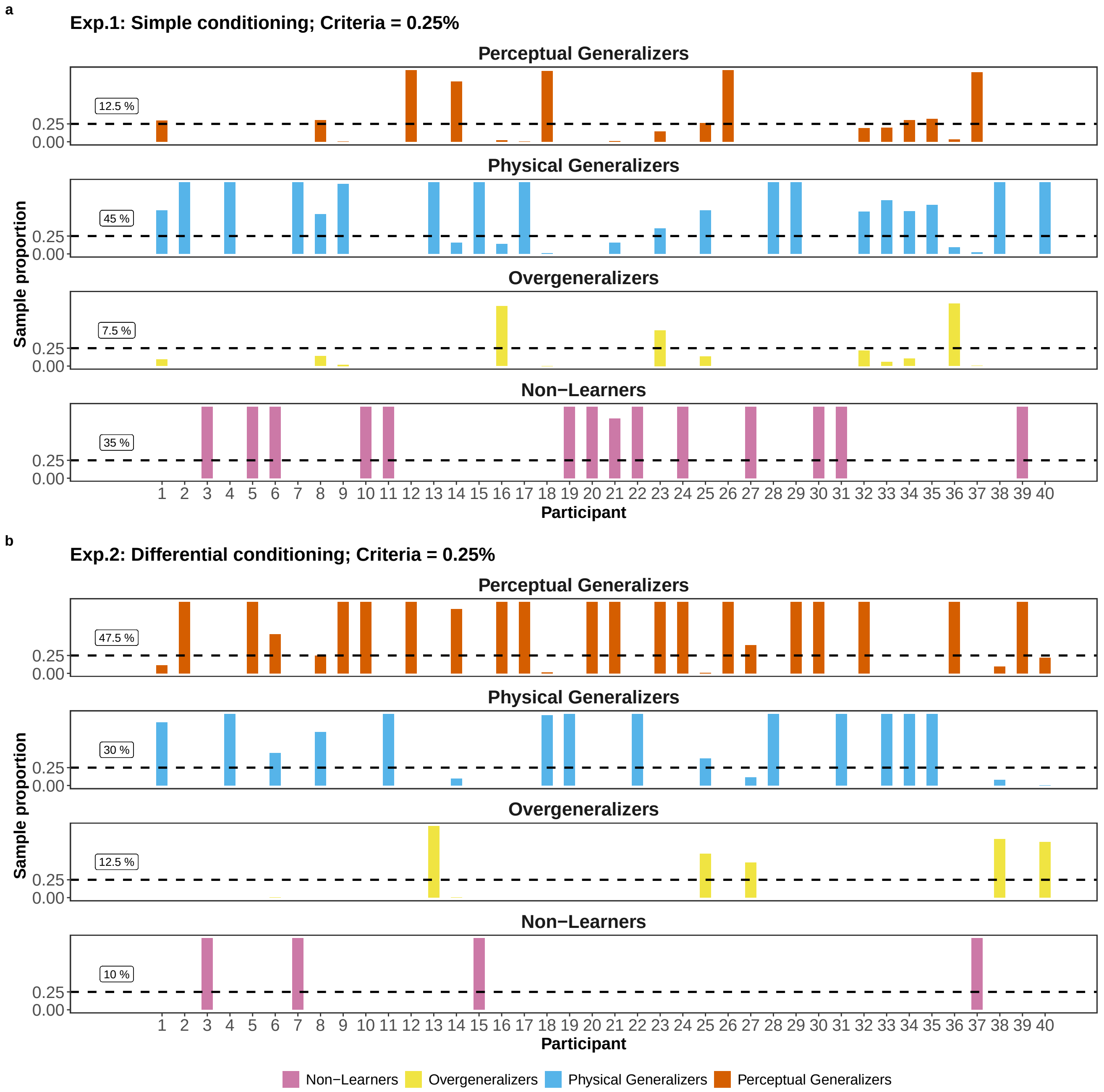

Supplementary Figure 5. The results of group allocation based on the criteria that the most frequent sampling value of the membership parameter m takes more than 25% of the posterior samples.

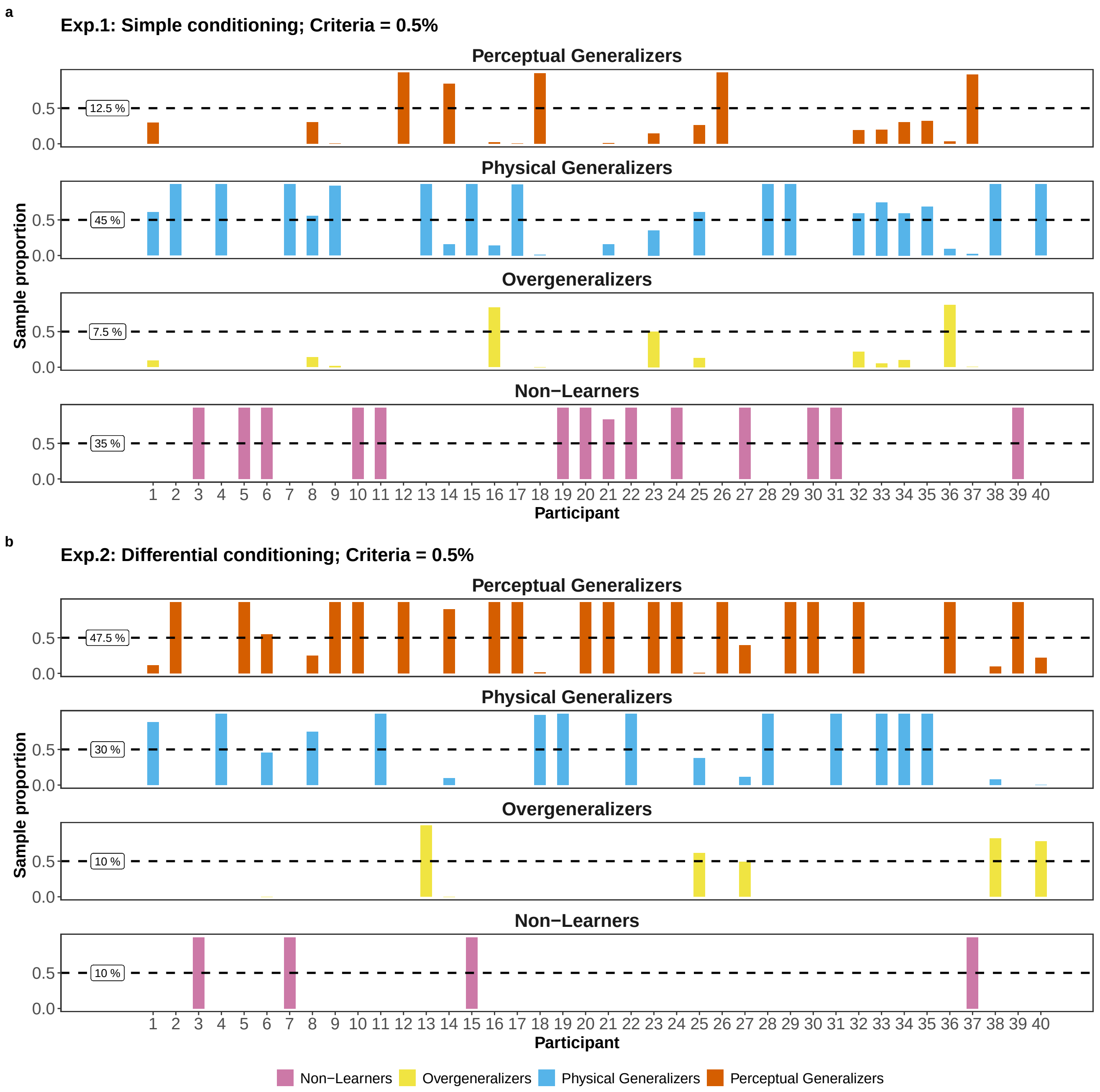

Supplementary Figure 6. The results of group allocation based on the criteria that the most frequent sampling value of the membership parameter m takes more than 50% of the posterior samples.

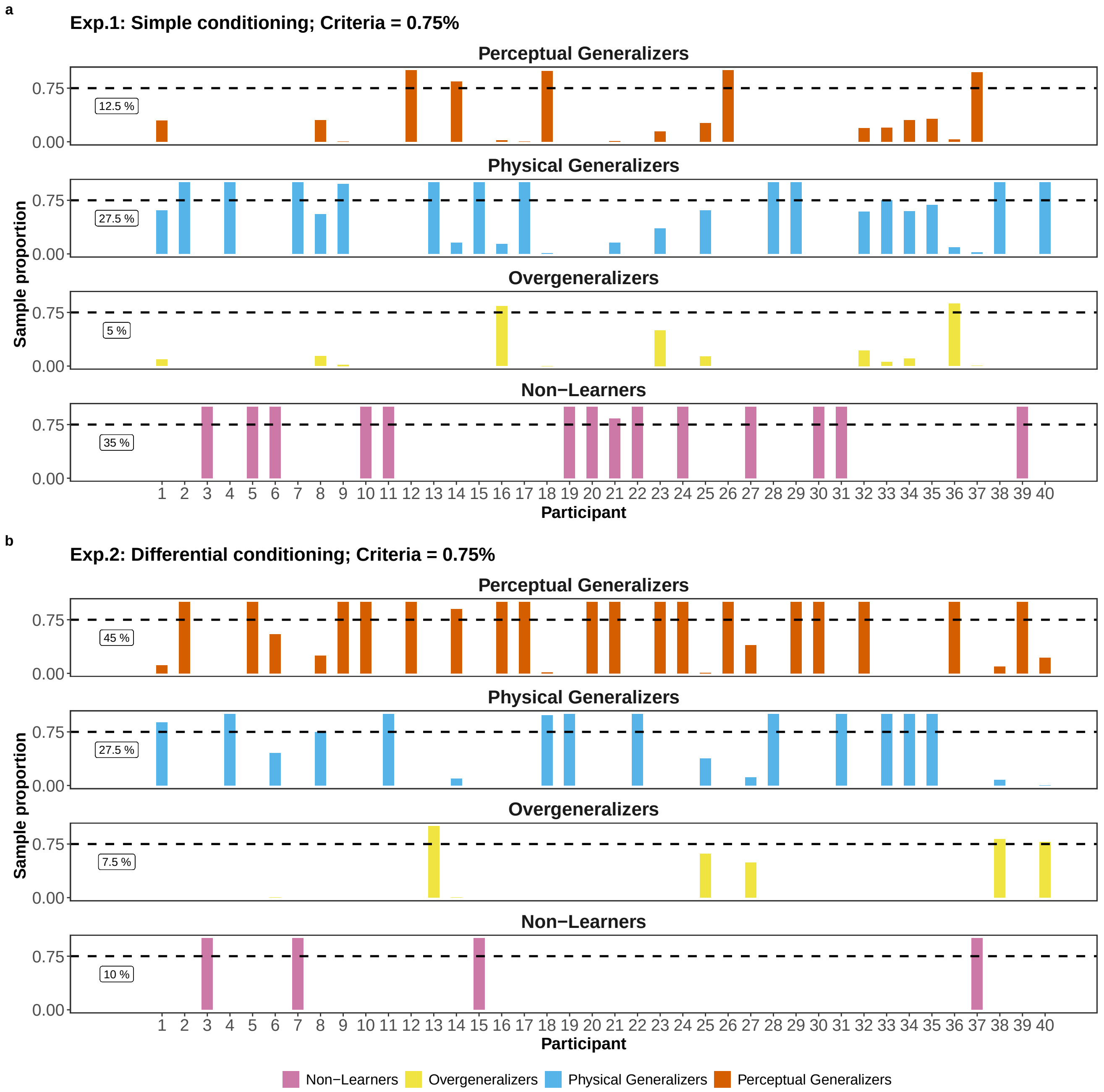

Supplementary Figure 7. The results of group allocation based on the criteria that the most frequent sampling value of the membership parameter m takes more than 75% of the posterior samples.

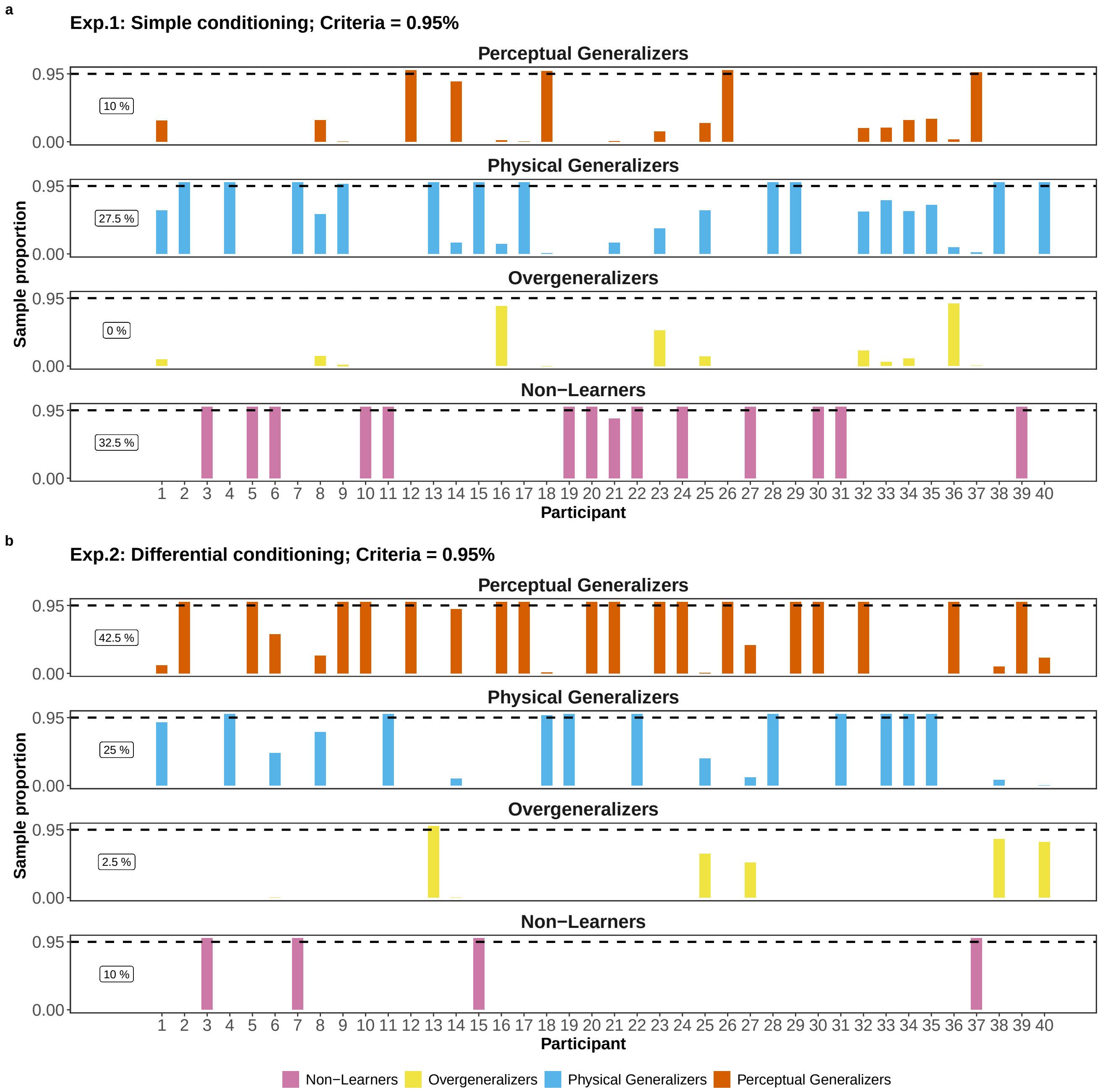

Supplementary Figure 8. The results of group allocation based on the criteria that the most frequent sampling value of the membership parameter m takes more than 95% of the posterior samples.

Participants are allocated to different groups based on the posterior samples of the group membership variable m_i (which can take integer values 1 to 4). Table 1 compares the group allocation result of a stricter criterion (i.e., 75% m_i samples on the same value else labeled unknown) with a more lenient criterion that allocates participants to one of the four latent groups based on the most frequent sampling value of m_i (i.e., maximum probability). To account for this uncertainty, we used the result of the strict criteria in the final analysis.

As expected, due to the inclusion of a safety cue (CS-) and a higher reinforcement rate, fewer *Non-Learners* were identified in Experiment 2 compared to Experiment 1. This implies that less stimulus-driven and more random behavior was observed in Experiment 1. As for individuals who exhibit some learning and similarity-based generalization patterns in Experiment 1, more participants were identified as *Physical Generalizers* than *Perceptual Generalizers*. In the second experiment, a similar proportion of participants in the *Overgeneralizers* group (7.5%) was observed. However, a different pattern concerning the generalization dimension was found: more people (45%) were classified as *Perceptual Generalizers* than *Physical Generalizers* (27.5%).

	Experiment 1		Experiment 2	
	Strict criteria	Lenient criteria	Strict criteria	Lenient criteria
Non-Learners	35%	35%	10%	10%
Overgeneralizers	5%	7.5%	7.5%	12.5%
Physical Generalizers	27.5%	45%	27.5%	30%
Perceptual Generalizers	12.5%	12.5%	45%	47.5%
Unknown	20%	0%	10%	0%

Supplementary Table 1. Percentage of model-based group allocation with both strict and flexible criteria. The strict criteria labels participants as unknown if none of the group allocation variable m_i comprises greater than 75% of posterior samples, and the lenient criteria allocates participants to either of the four latent groups based on the most frequent sampling value.

Supplementary Note 4

In the main manuscript (Figure 4), we have demonstrated, through posterior predictive checks, that the model can effectively capture and fit the observed generalization data. In this section, we present additional results showcasing the mean posterior predictive checks at the individual level for two experimental data sets. These results serve to further illustrate that the model performs well in accurately fitting the data on an individual level.

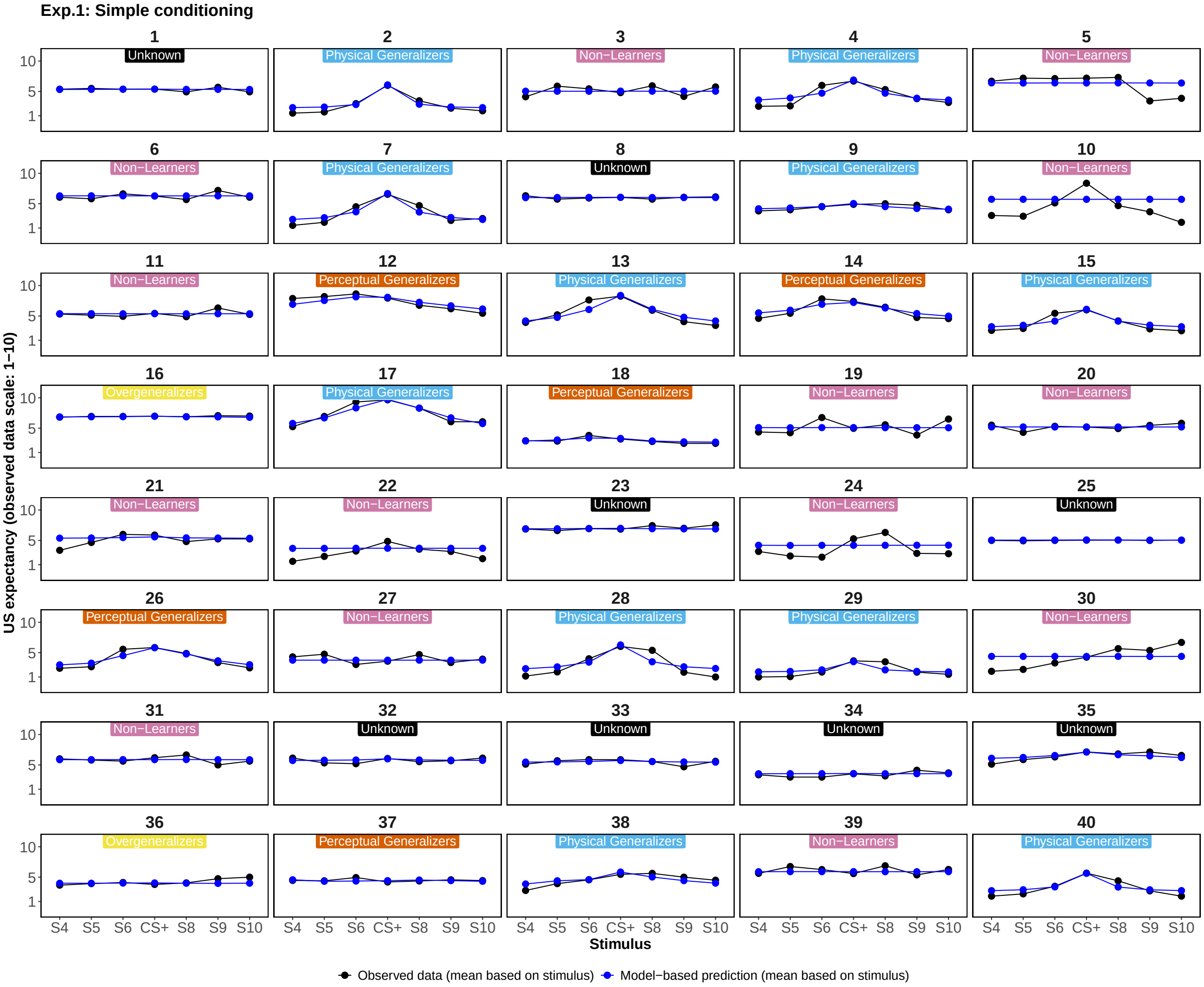

Supplementary Figure 9. Posterior predictive checks with the mean of individual observed generalization data for Experiment 1.

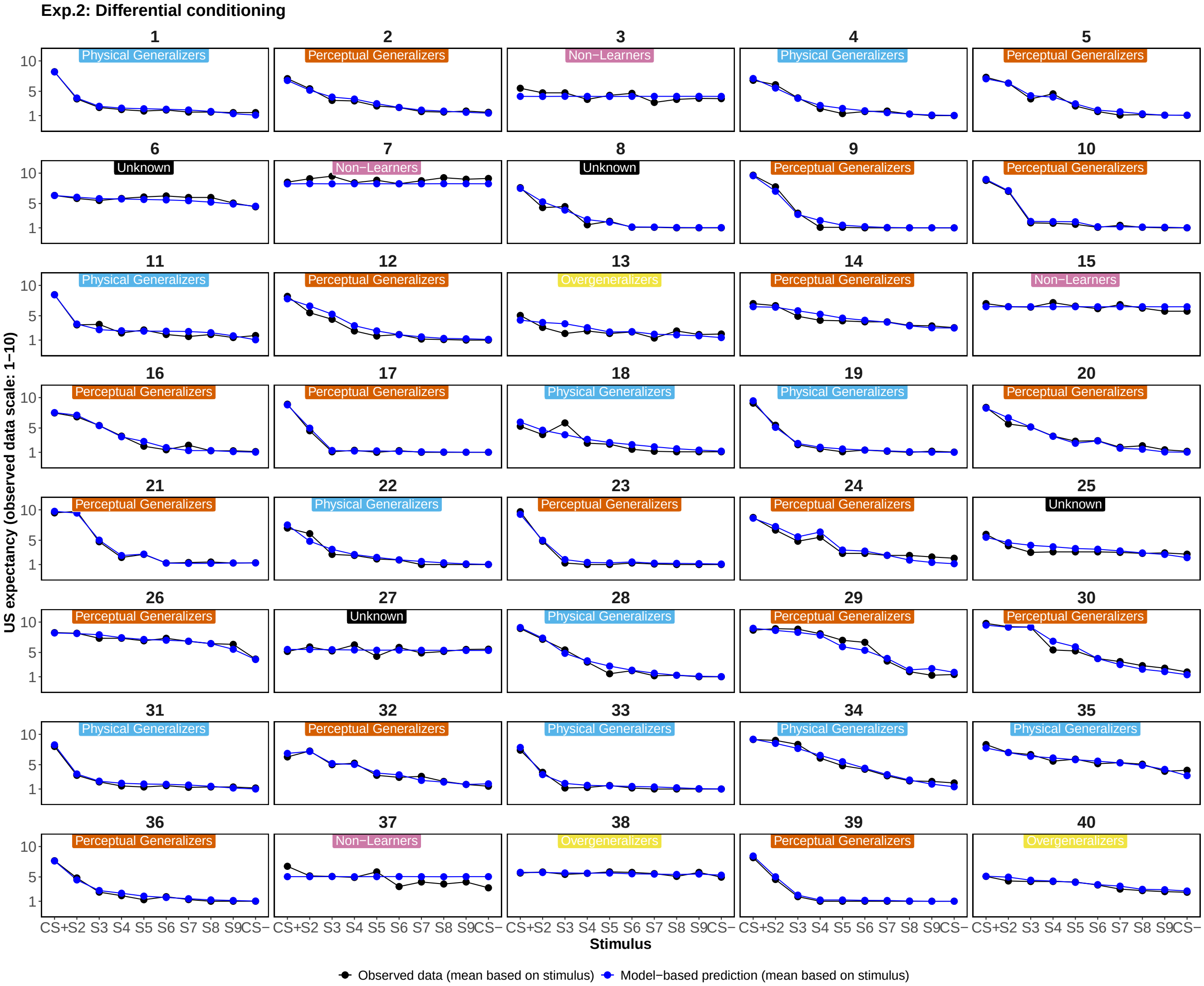

Supplementary Figure 10. Posterior predictive checks with the mean of individual observed generalization data for Experiment 2.

Supplementary Note 5

Four MCMC chains were run for the model, with 100000 iterations, 75000 burn-ins, and a thinning factor of 10 for each chain (i.e., only each 10-th sample was retained). This returns 10000 samples in total for each parameter. Here we show the trace plots of the person-specific learning rate α, the generalization rate λ, the base line response parameter $w0$, and the scaling parameter $w1$. For the group level, we show the trace plots of α_{μ}, λ_{μ}, the response noise σ, and the group probability p. The parameters without well mixing chains are the indication of failing convergence. Additionally, we checked the $\hat{R}$ value of Gelman and Rubin diagnostics for convergence. The $\hat{R}$ value close to 1 is deemed to represent stationary distributions. According to the parameter trace plots and $\hat{R}$ values, Participant 32 in Experiment 1 and Participant 8 and 25 in Experiment 2 have convergence problems. With the strict group allocation criteria (75% posterior samples of group membership are the same value), these participants are labelled as *Unknown* and were not include in the analysis.

MCMC traceplots

Group-level parameter

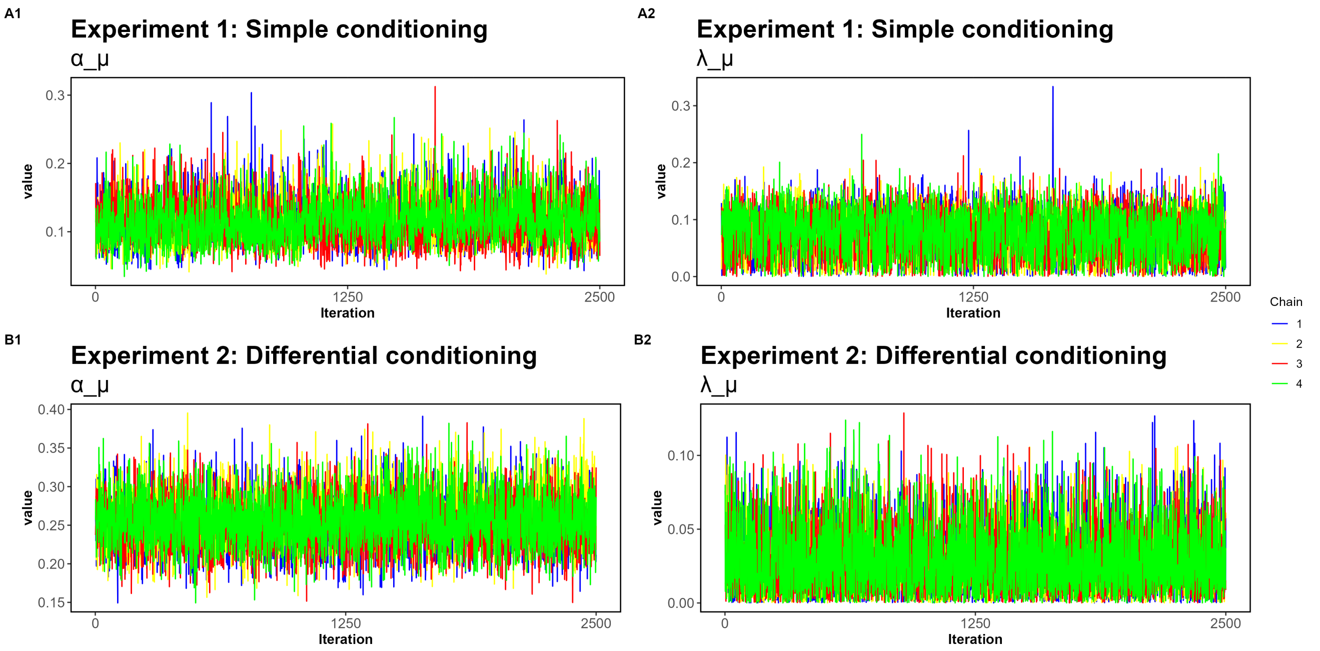

Supplementary Figure 11. The trace plots for the group mean learning rate α_μ and generalization rate λ_μ of Experiment 1 and 2.

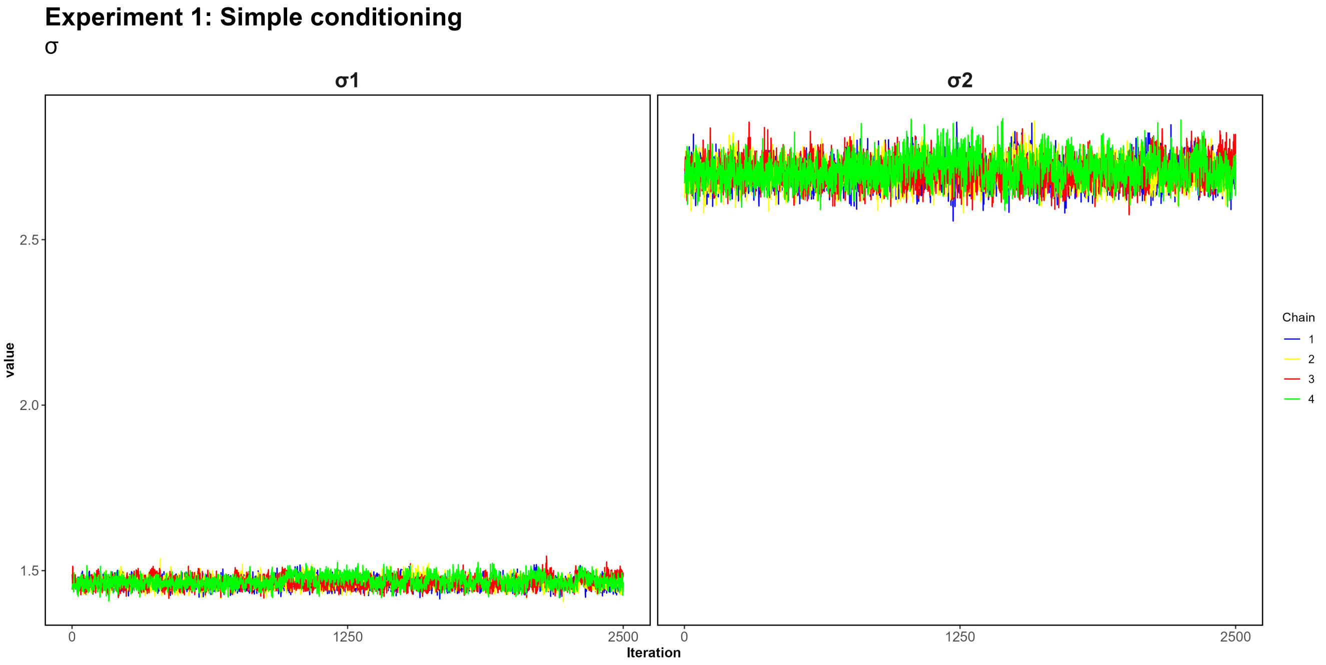

Supplementary Figure 12. The trace plots for the response noise parameter σ of Experiment 1.

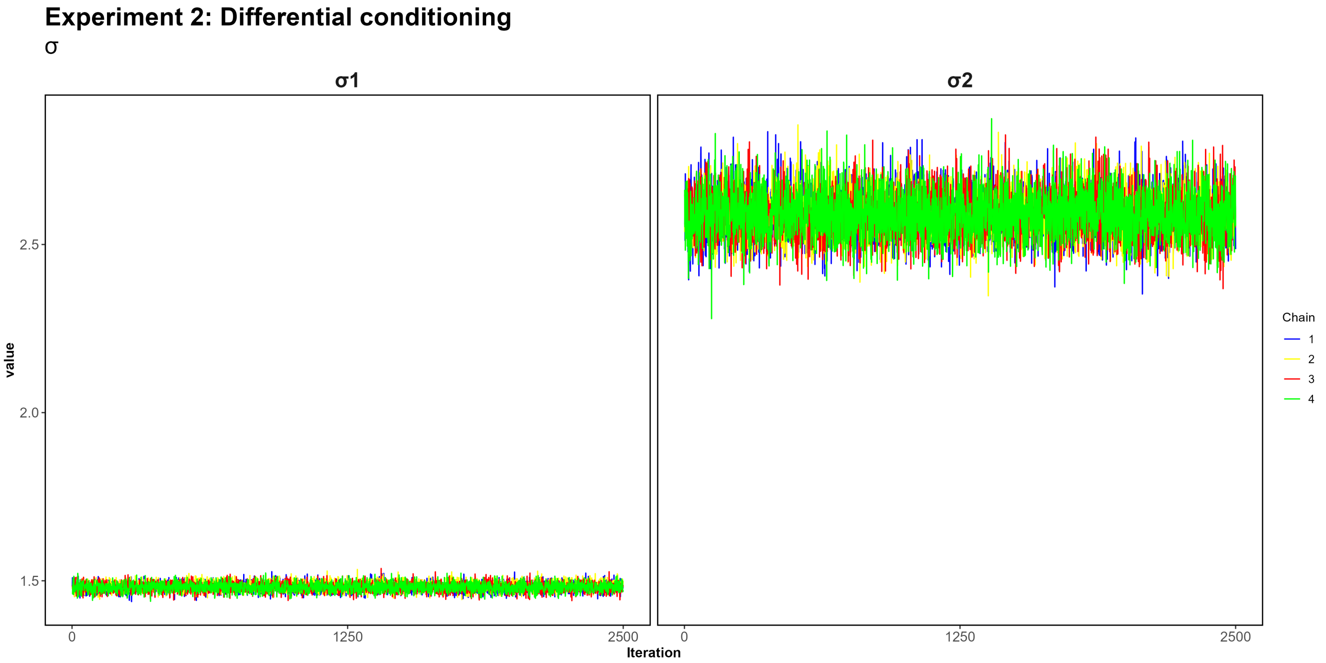

Supplementary Figure 13. The trace plots for the response noise parameter σ of Experiment 2.

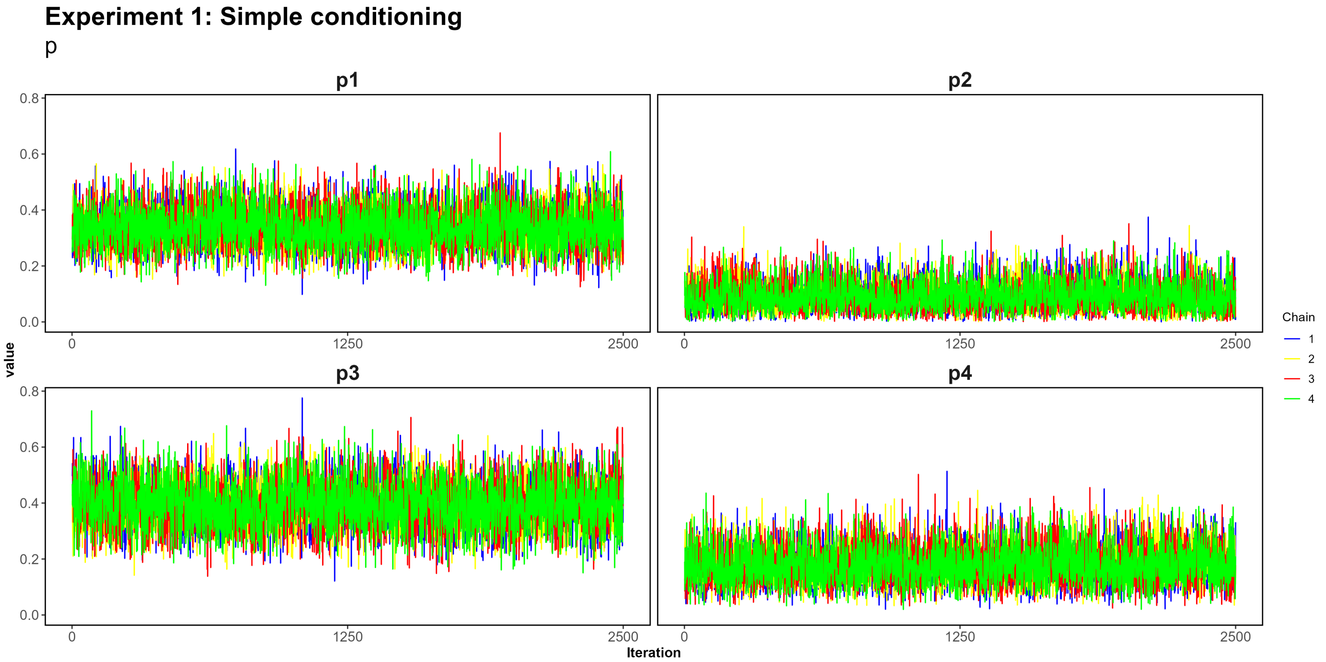

Supplementary Figure 14. The trace plots for the probability membership parameter p of Experiment 1. p1: *Non-Learners*; p2: *Overgeneralizers*; p3: *Physical Generalizers*; p4: *Perceptual Generalizers*.

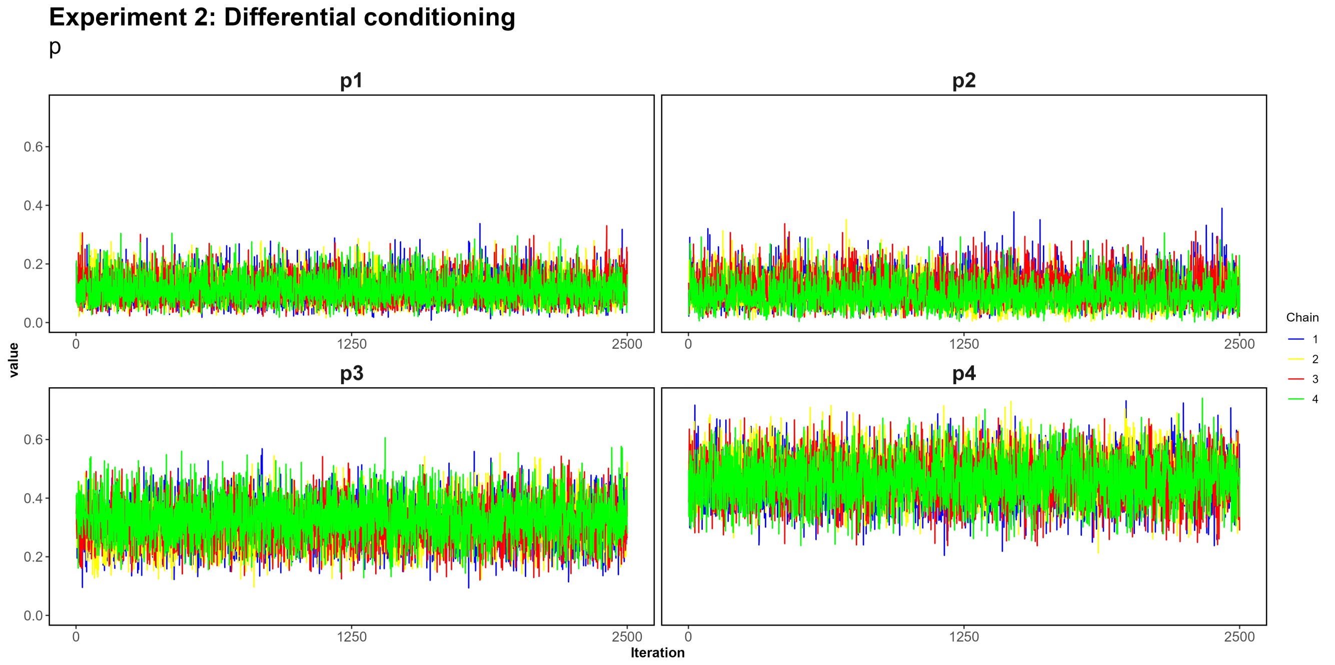

Supplementary Figure 15. The trace plots for the probability membership parameter p of Experiment 2. p1: *Non-Learners*; p2: *Overgeneralizers*; p3: *Physical Generalizers*; p4: *Perceptual Generalizers*.

Person-specific parameter

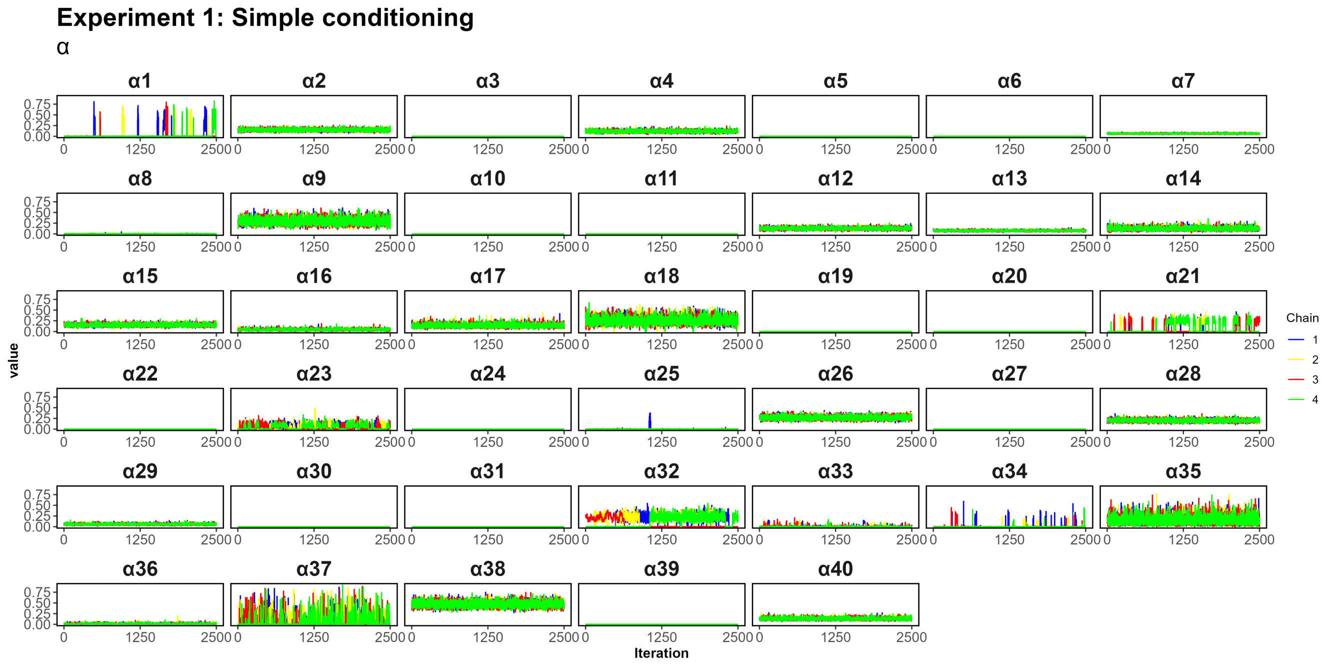

Supplementary Figure 16. The trace plot for person-specific learning rates α in Experiment 1.

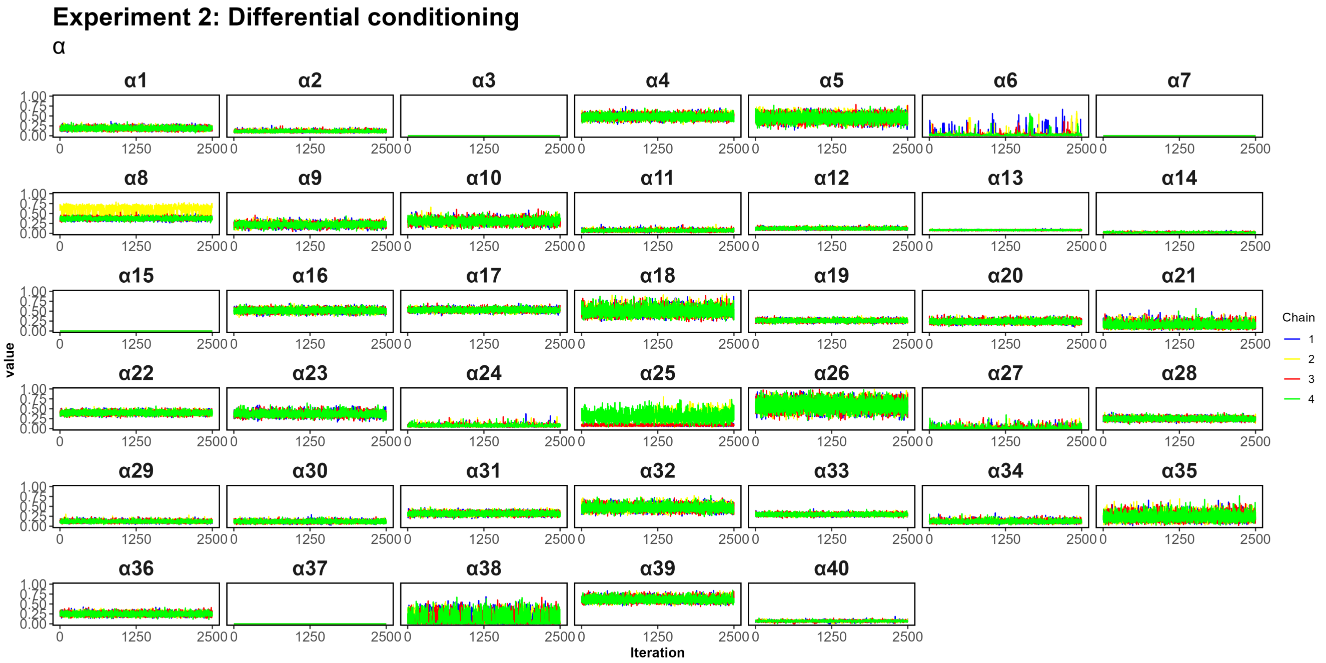

Supplementary Figure 17. The trace plot for person-specific learning rate α in Experiment 2.

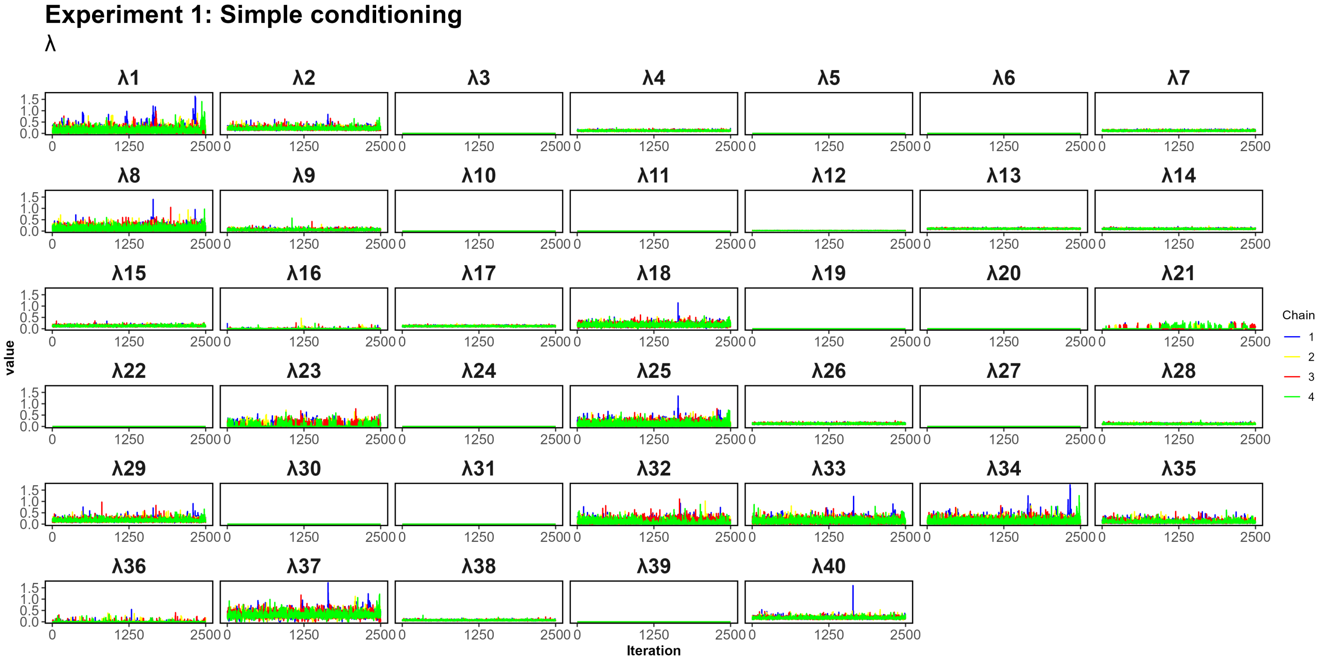

Supplementary Figure 18. The trace plot for person-specific learning rate λ in Experiment 1.

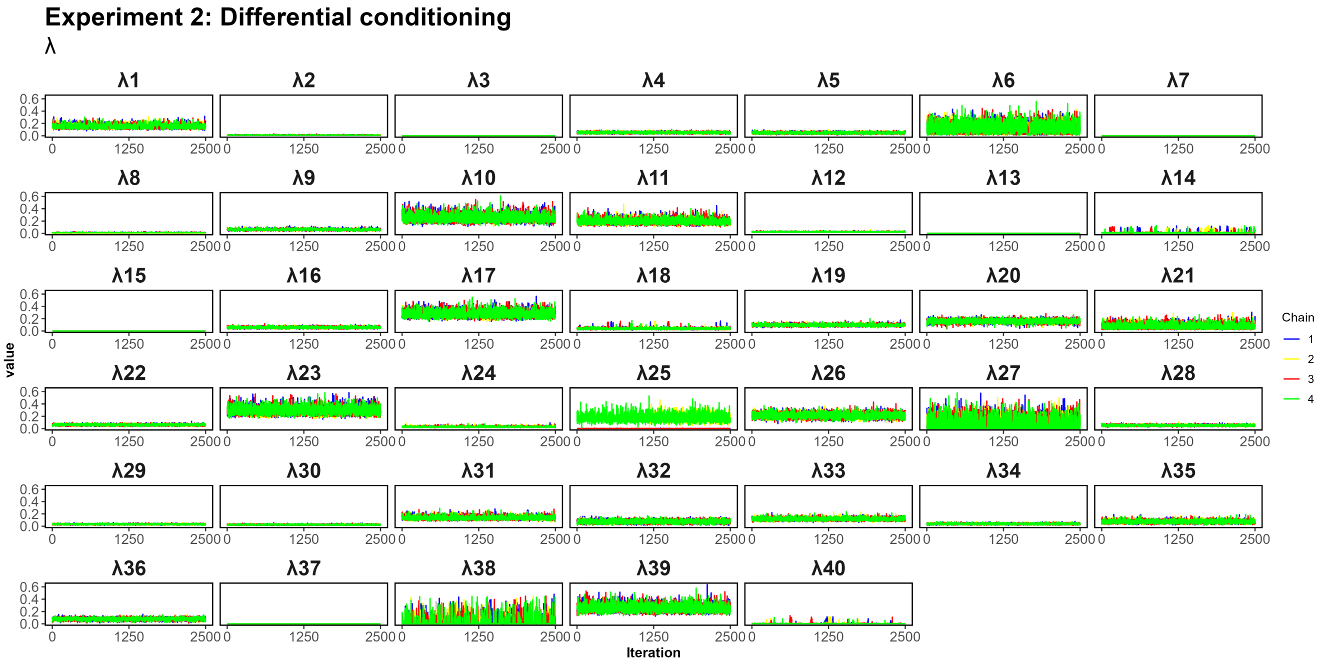

Supplementary Figure 19. The trace plot for person-specific learning rate λ in Experiment 2.

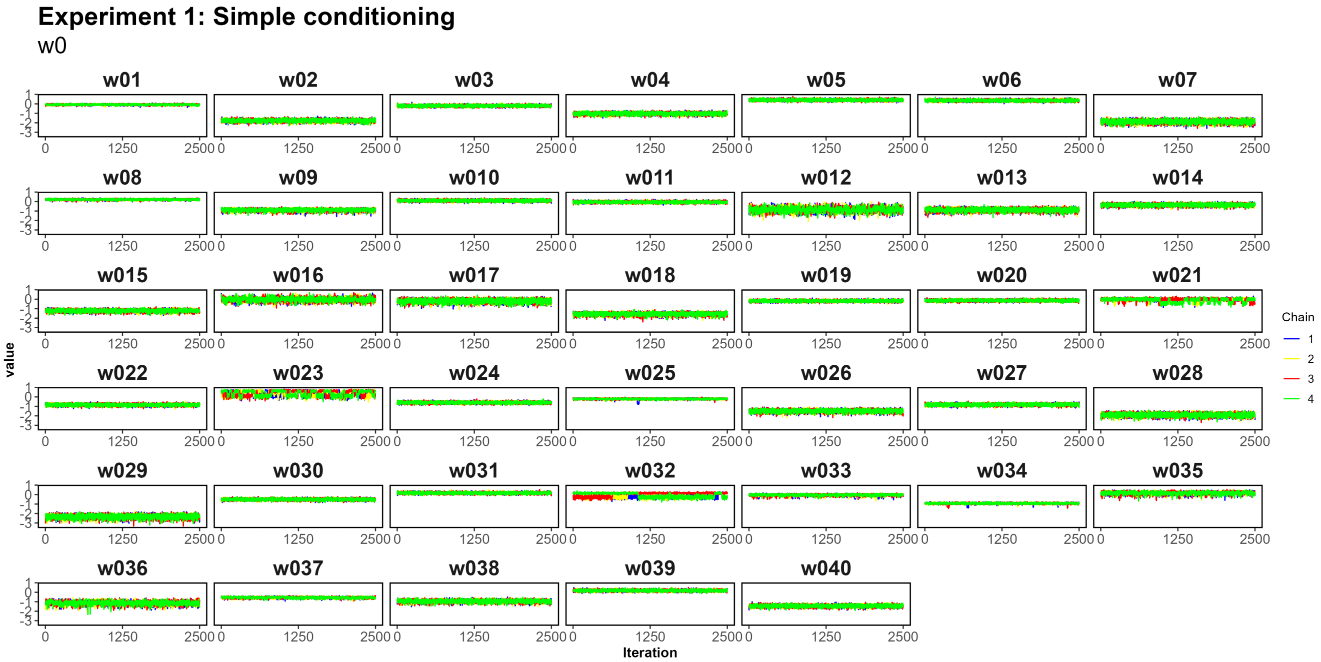

Supplementary Figure 20. The trace plot for person-specific learning rate $w0$ in Experiment 1.

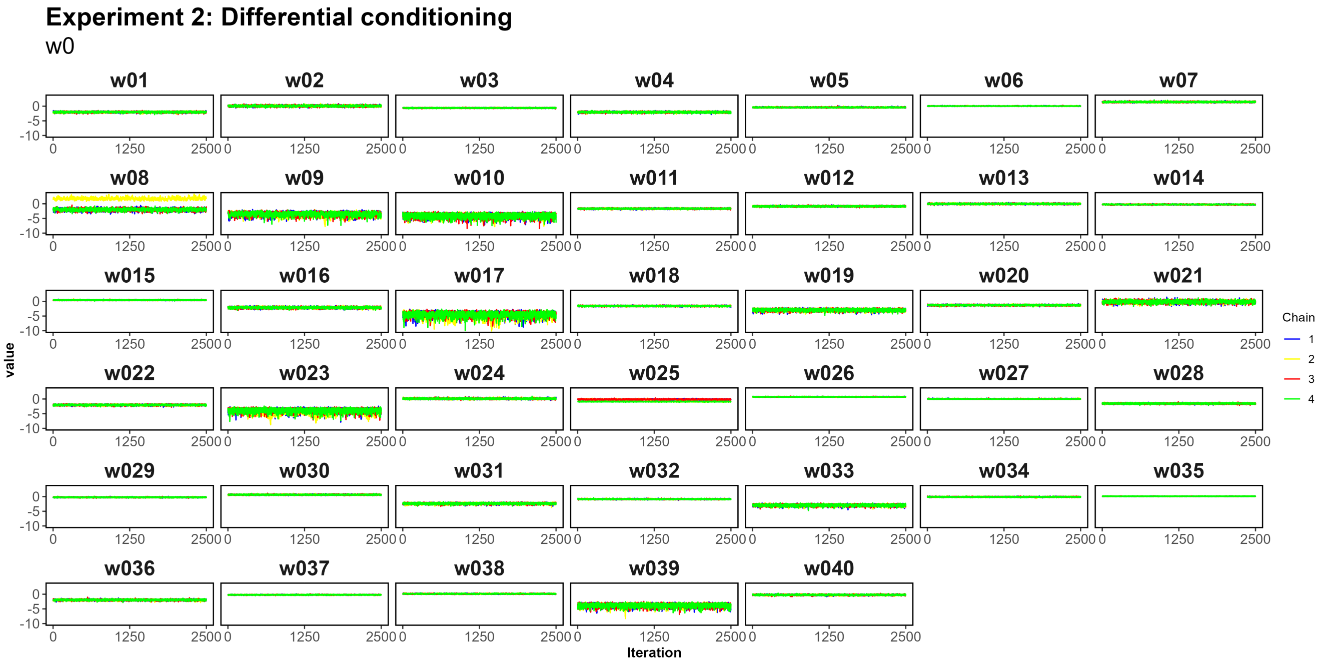

Supplementary Figure 21. The trace plot for person-specific learning rate $w0$ in Experiment 2.

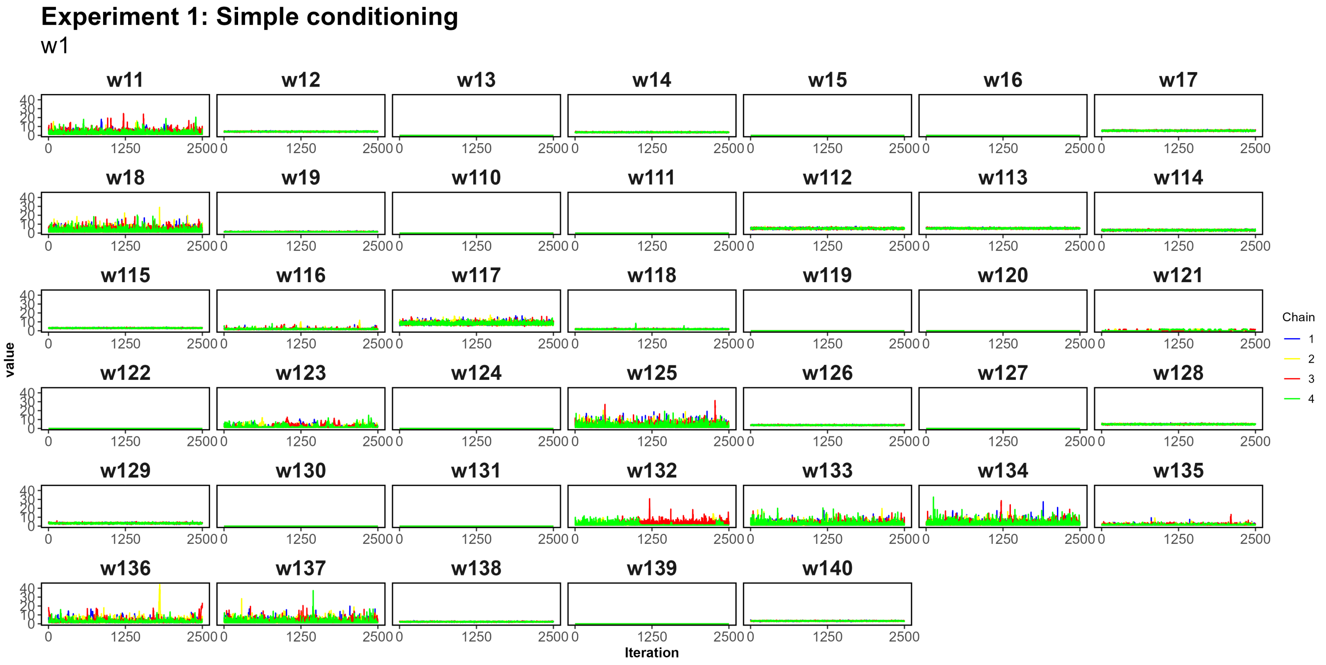

Supplementary Figure 22. The trace plot for person-specific learning rate $w1$ in Experiment 1.

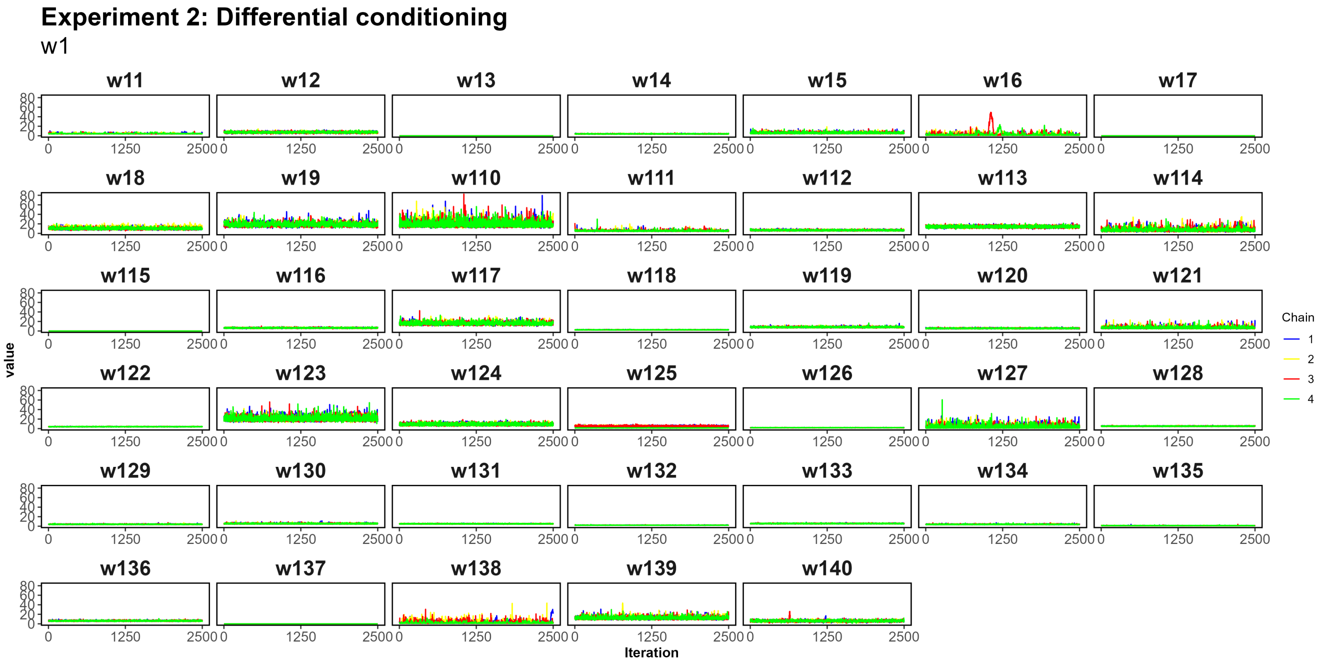

Supplementary Figure 23. The trace plot for person-specific learning rate $w1$ in Experiment 2.

Gelman and Rubin diagnostics

Gelman and Rubin diagnostics[1,2] are based on evaluating variances within and between several simulated MCMC chains. A statistically significant divergence between the two variances indicates non-convergence.

Group-level parameter

α_μ	λ_μ	$\sigma[1]$	$\sigma[2]$	$p[1]$	$p[2]$	$p[3]$	$p[4]$
1.001723	1.001365	1.014066	1.008066	1.000016	1.001448	1.000206	1.001789

Supplementary Table 2. $\hat{R}$ values of group-level learning rate α_μ, generalization rate λ_μ, response noise parameter σ and membership probability p of Experiment 1.

α_μ	λ_μ	$\sigma[1]$	$\sigma[2]$	$p[1]$	$p[2]$	$p[3]$	$p[4]$
1.017304	1.000675	1.016618	1.000073	0.9999526	1.034891	1.025277	1.016461

Supplementary Table 3. $\hat{R}$ values of group-level learning rate α_μ, generalization rate λ_μ, response noise parameter σ and membership probability p of Experiment 2.

Person-specific parameter

Participant	α	λ	w0	w1
1	1.037537	1.0181240	1.0004197	1.009737
2	1.000397	1.0009649	1.0006419	1.001120
3	NA	NA	0.9999854	NA
4	1.000661	1.0002961	1.0005607	1.000762
5	NA	NA	1.0002634	NA
6	NA	NA	1.0001703	NA
7	1.000066	1.0003887	1.0005349	1.000570
8	1.015622	1.0021061	1.0002576	1.004444
9	1.000274	1.0027240	1.0024690	1.003731
10	NA	NA	1.0001317	NA
11	NA	NA	1.0000414	NA
12	1.000025	1.0001788	1.0006824	1.000429
13	1.000103	0.9999999	1.0002187	1.000370
14	1.000225	1.0001521	1.0001413	1.001479
15	1.000474	1.0000077	1.0000035	1.000415
16	1.002384	1.0303263	1.0018929	1.033697
17	1.000124	1.0014379	1.0014905	1.003450
18	1.000422	1.0004453	1.0006287	1.006575
19	NA	NA	1.0000218	NA
20	NA	NA	1.0000032	NA
21	1.071919	1.0621482	1.0515870	1.076677
22	NA	NA	0.9999973	NA
23	1.005581	1.0022232	1.0040776	1.017478
24	NA	NA	1.0001112	NA
25	1.286468	1.0020787	1.0140726	1.001562
26	1.000374	1.0000348	1.0004591	1.000210
27	NA	NA	0.9999130	NA
28	1.000240	1.0003939	1.0010696	1.000970
29	1.000414	1.0028677	0.9999089	1.000285
30	NA	NA	1.0001700	NA
31	NA	NA	0.9999149	NA
32	1.059145	1.0148040	1.0537948	1.069951
33	1.014843	1.0011527	1.0025014	1.005764
34	1.097830	1.0106833	1.0071754	1.004535
35	1.001595	1.0006074	1.0034937	1.032400
36	1.006841	1.0122919	1.0014418	1.042496
37	1.000322	1.0049800	1.0006557	1.001966
38	1.000381	1.0010006	1.0011979	1.001215
39	NA	NA	1.0003823	NA
40	1.000031	1.0097482	1.0011983	1.000888

Supplementary Table 4. $\hat{R}$ values of person-specific learning rate α, generalization rate λ, base-line response parameter w0 and scaling parameter w1 in Experiment 1

Participant	α	λ	w0	w1
1	1.0005310	1.0020090	1.0000280	1.007945
2	1.0011005	1.0013360	1.0001300	1.000522
3	NA	NA	1.0000896	NA
4	0.9999599	1.0001296	1.0001635	1.000257
5	1.0007406	1.0006212	1.0001727	1.000628
6	1.0228074	1.0014180	1.0012417	1.158448
7	NA	NA	0.9998524	NA
8	3.0471530	1.3554996	6.5573700	1.179697
9	1.0078804	1.0137895	1.0164488	1.004339
10	1.0007042	1.0078448	1.0051424	1.002340
11	1.0009400	1.0003764	1.0004453	1.000981
12	1.0001129	1.0005234	1.0006593	1.000437
13	0.9999441	1.0001211	1.0001612	1.000488
14	1.0041575	1.0119128	1.0040298	1.005268
15	NA	NA	0.9998918	NA
16	1.0002246	1.0006283	1.0000149	1.000308
17	1.0025096	1.0414517	1.0476077	1.016508
18	1.0015529	1.0083565	1.0000329	1.001994
19	1.0002772	0.9999491	1.0007059	1.000579
20	1.0001201	1.0009037	1.0001247	1.000101
21	1.0001005	1.0004704	1.0001740	1.000659
22	1.0002841	0.9999485	1.0005675	1.001145
23	1.0111584	1.2514619	1.2154431	1.004573
24	1.0015882	1.0031667	1.0007643	1.000307
25	1.8212368	2.2331964	2.3638625	2.146215
26	1.0006198	1.0016268	1.0002796	1.000182
27	1.0022854	1.0014579	1.0006475	1.001506
28	0.9999259	1.0000808	1.0001436	1.000421
29	1.0003906	0.9998878	1.0004282	1.000028
30	1.0011807	1.0005517	1.0002118	1.001780
31	1.0002393	1.0000823	1.0005804	1.000372
32	1.0002487	1.0013153	1.0009423	1.002095
33	1.0004392	1.0004283	1.0001127	1.000118
34	1.0002214	1.0002975	0.9999199	1.000317
35	1.0003853	0.9998954	1.0001581	1.002592
36	1.0000541	1.0071532	1.0005655	1.002130
37	NA	NA	1.0000237	NA
38	1.0008902	1.0023355	1.0003590	1.033620
39	1.0062933	1.0209931	1.0019865	1.018636
40	1.0025201	1.0144177	1.0023968	1.006490

Supplementary Table 5. $\hat{R}$ values of person-specific learning rate α, generalization rate λ, base-line response parameter w0 and scaling parameter w1 of Experiment 2

From Figure 24 to 36, we present the plots that represent distribution of both group-level and individual-level parameters.

Group-level parameter

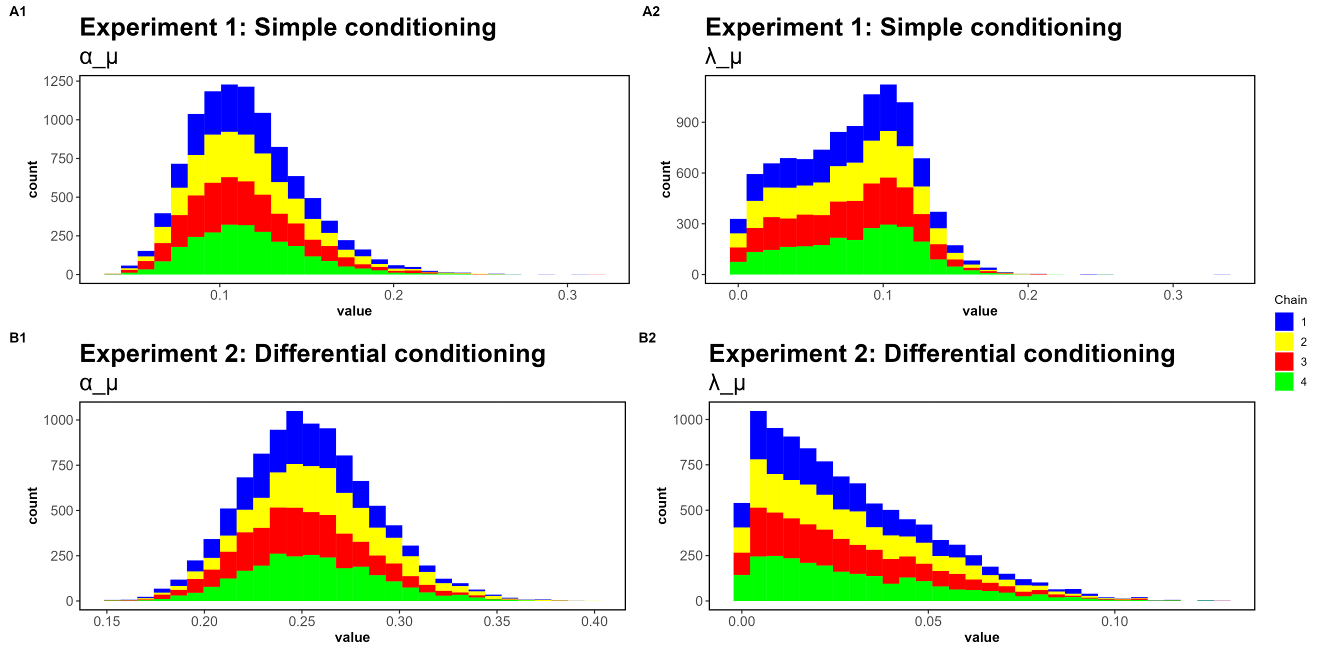

Supplementary Figure 24. The posterior distributions for the group mean learning rate α_μ and generalization rate λ_μ of Experiment 1 and 2.

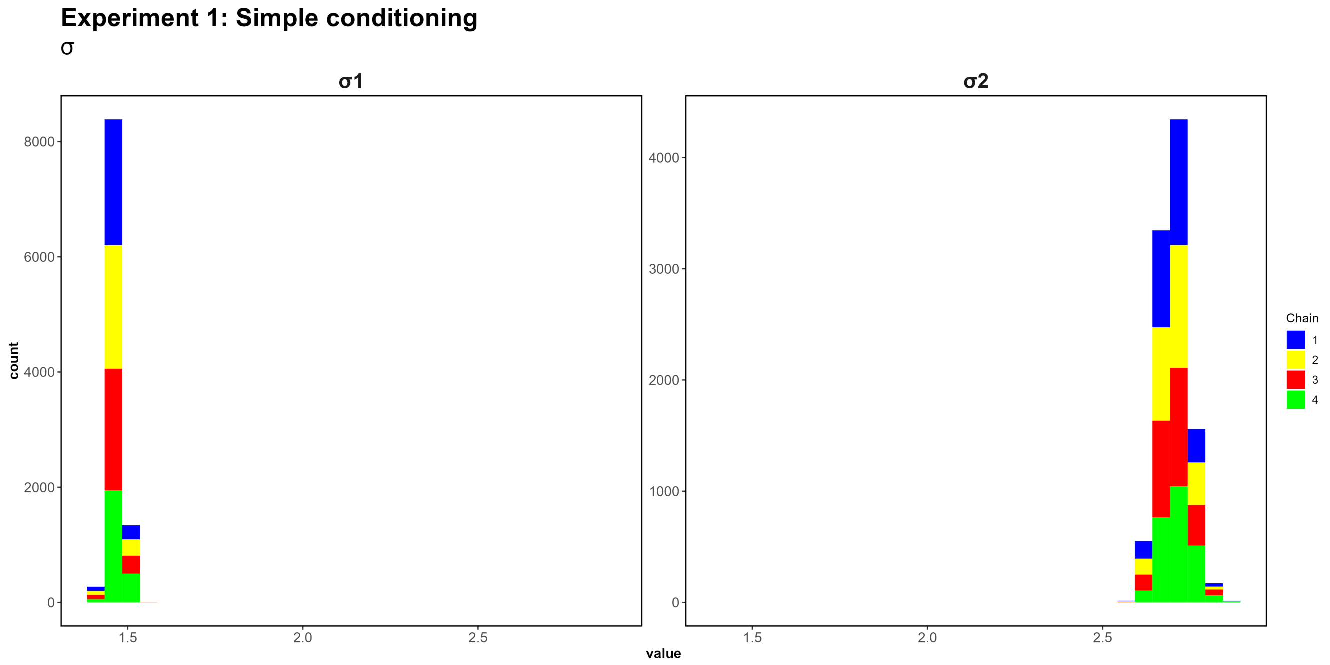

Supplementary Figure 25. The posterior distributions for the response noise parameter σ of Experiment 1.

Experiment 2: Differential conditioning

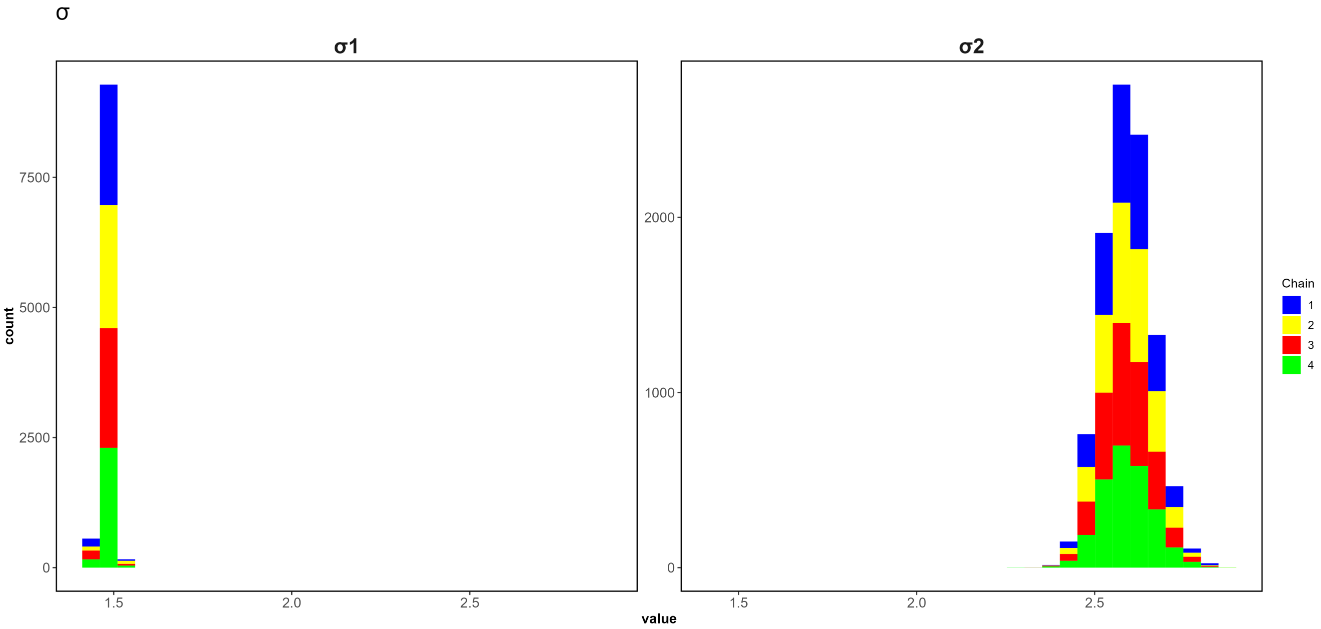

Supplementary Figure 26. The posterior distributions for the response noise parameter σ of Experiment 2.

Experiment 1: Simple conditioning

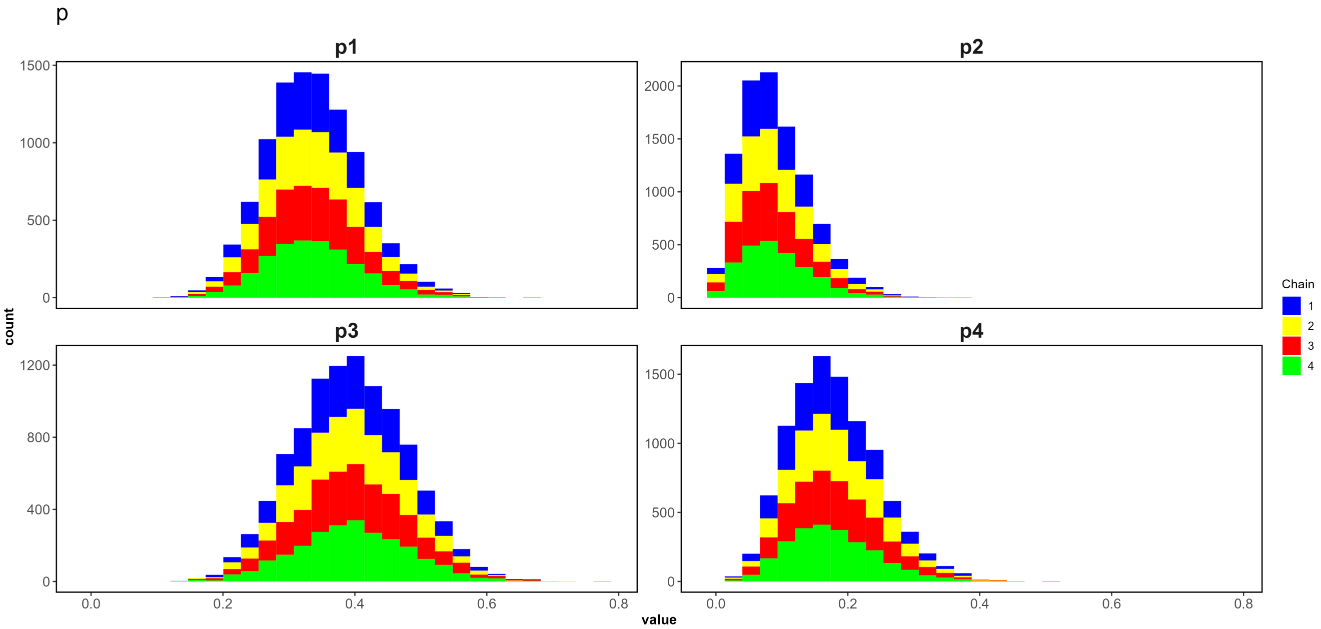

Supplementary Figure 27. The posterior distributions for the probability membership parameter p of Experiment 1. p1: *Non-Learners*; p2: *Overgeneralizers*; p3: *Physical Generalizers*; p4: *Perceptual Generalizers*.

Experiment 2: Differential conditioning

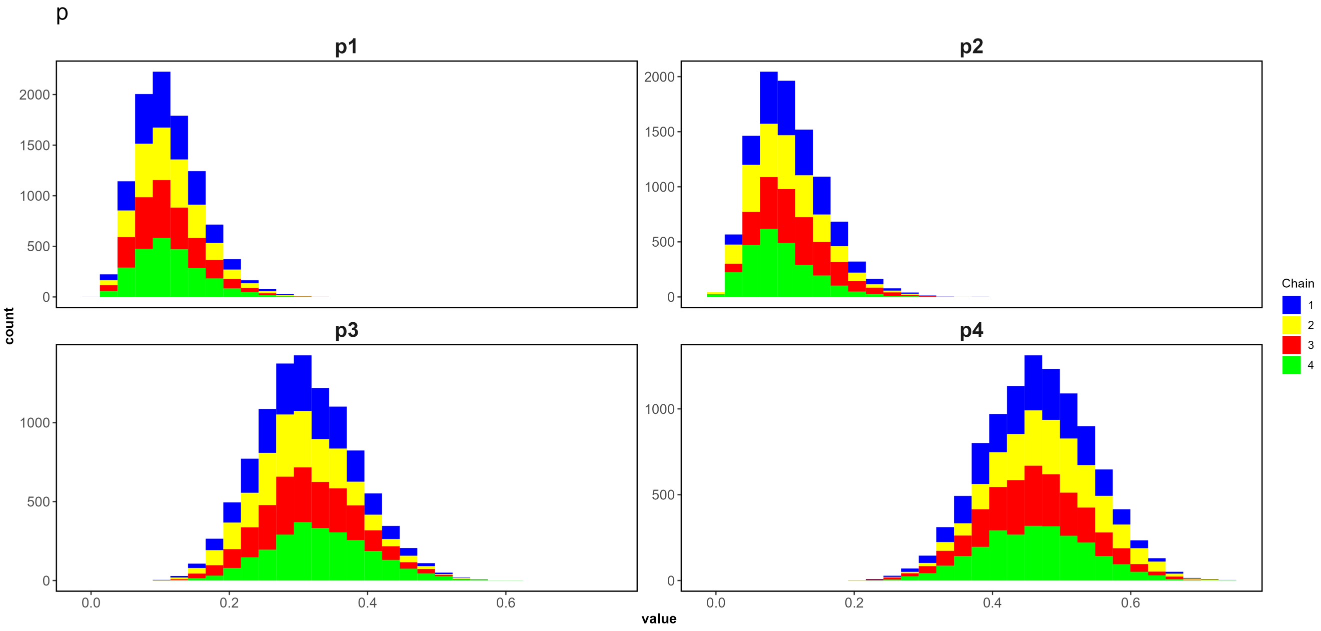

Supplementary Figure 28. The posterior distributions for the probability membership parameter p of Experiment 2. p1: *Non-Learners*; p2: *Overgeneralizers*; p3: *Physical Generalizers*; p4: *Perceptual Generalizers*.

Person-specific parameter

Experiment 1: Simple conditioning

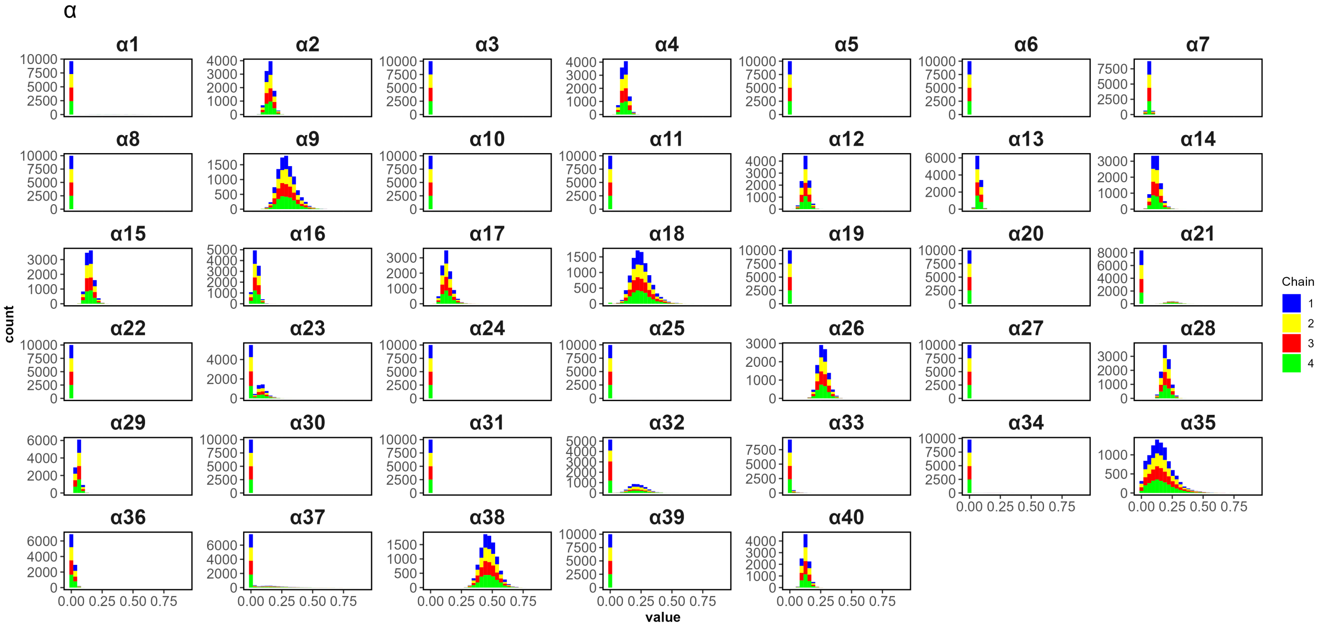

Supplementary Figure 29. The posterior distributions for person-specific learning rates α in Experiment 1.

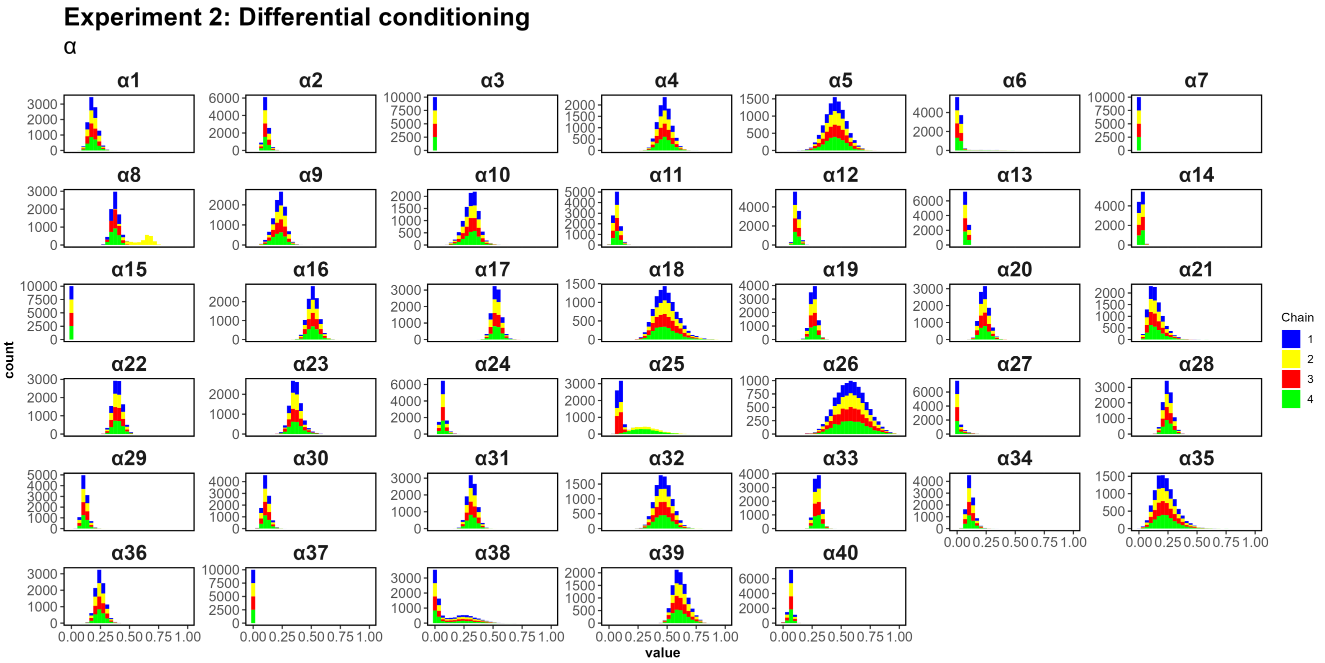

Supplementary Figure 30. The posterior distributions for person-specific learning rate α in Experiment 2.

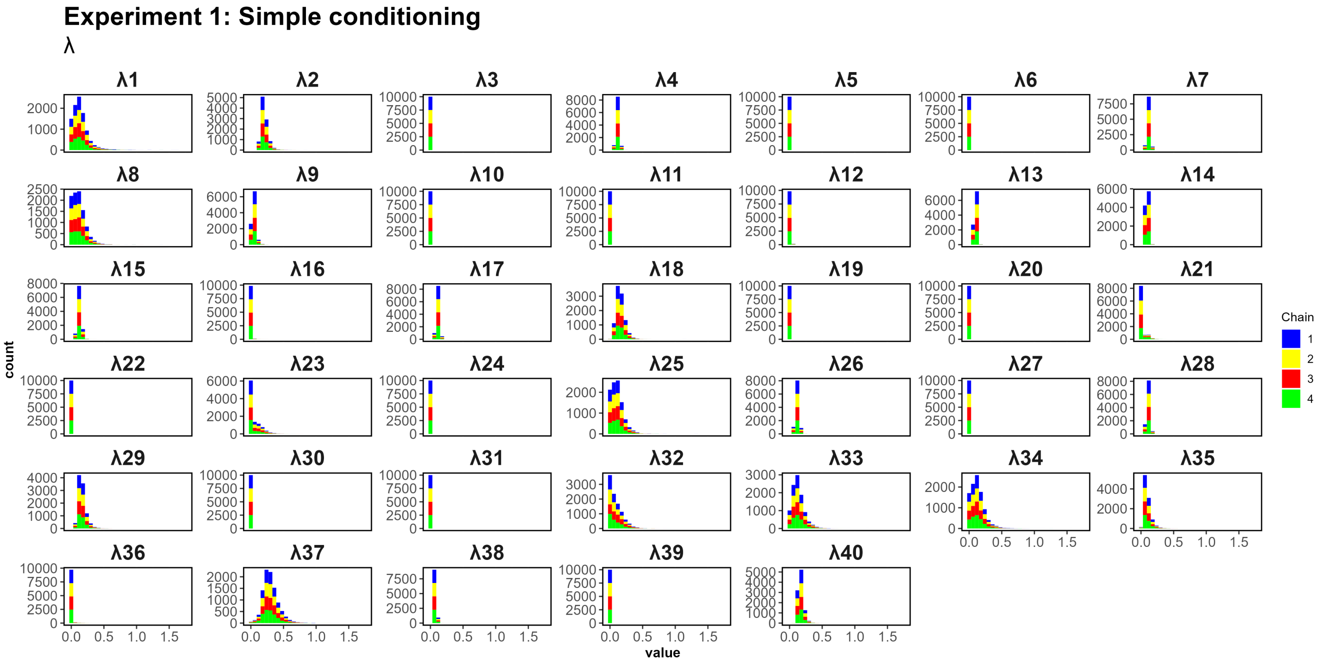

Supplementary Figure 31. The posterior distributions for person-specific learning rate λ in Experiment 1.

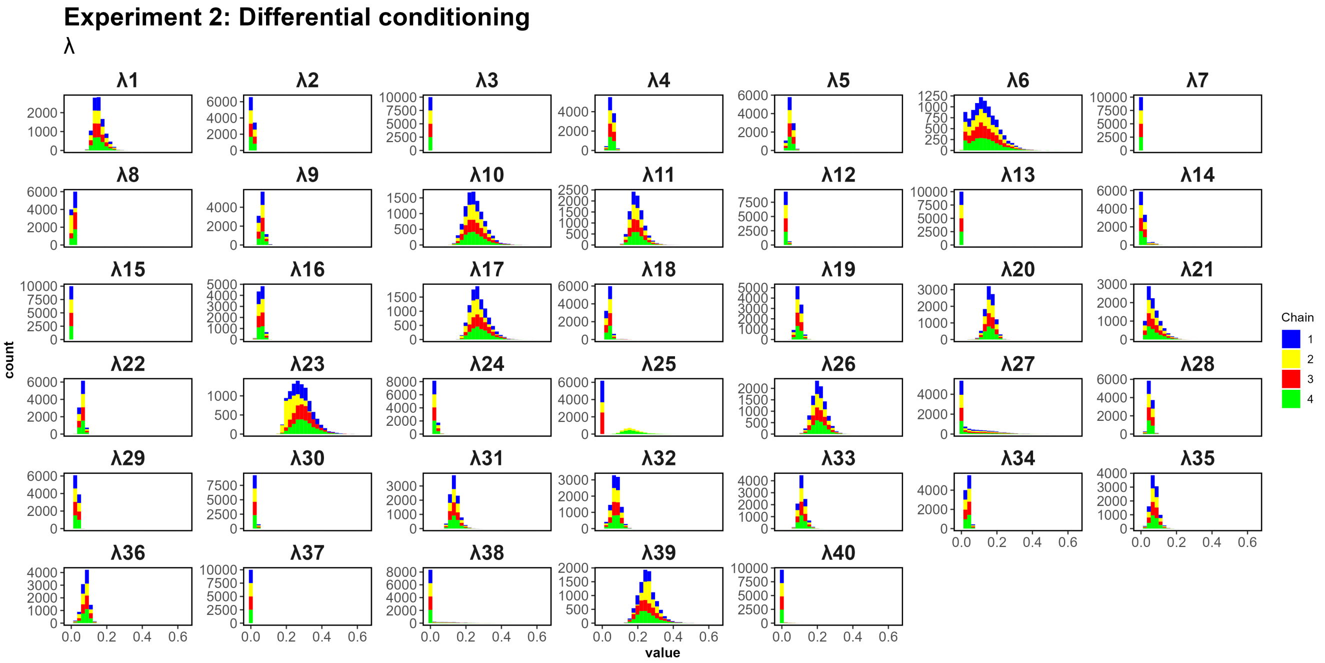

Supplementary Figure 32. The posterior distributions for person-specific learning rate λ in Experiment 2.

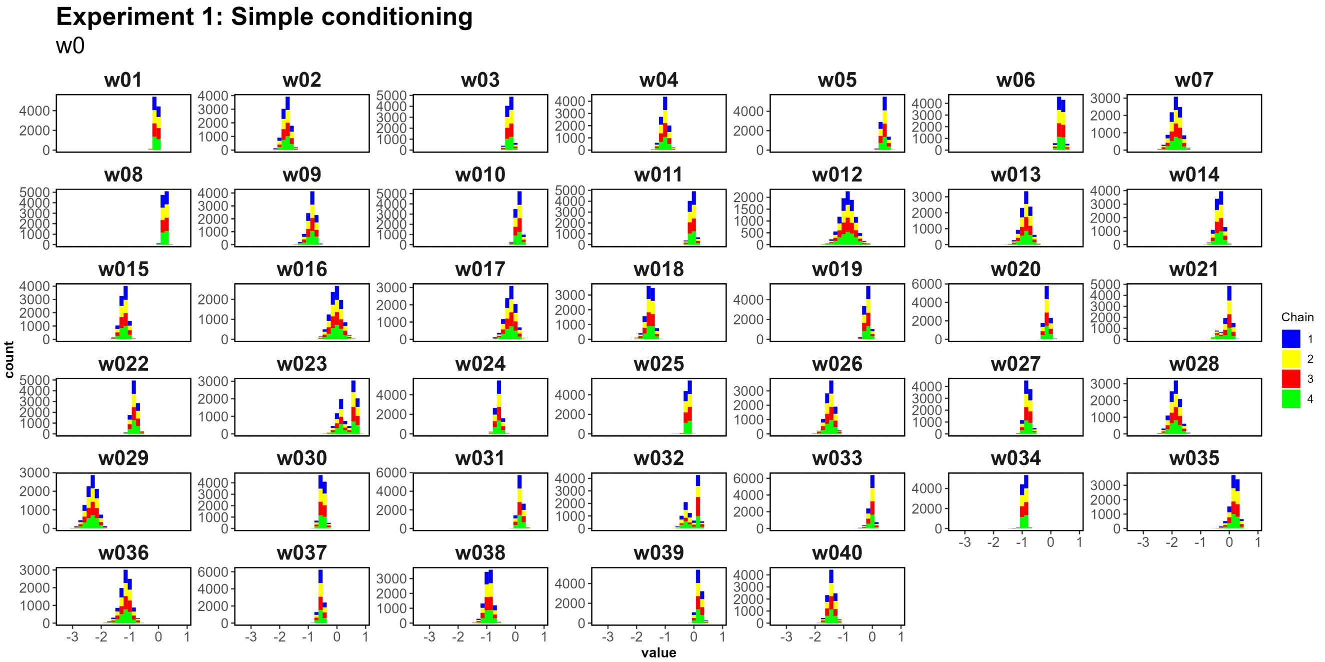

Supplementary Figure 33. The posterior distributions for person-specific learning rate $w0$ in Experiment 1.

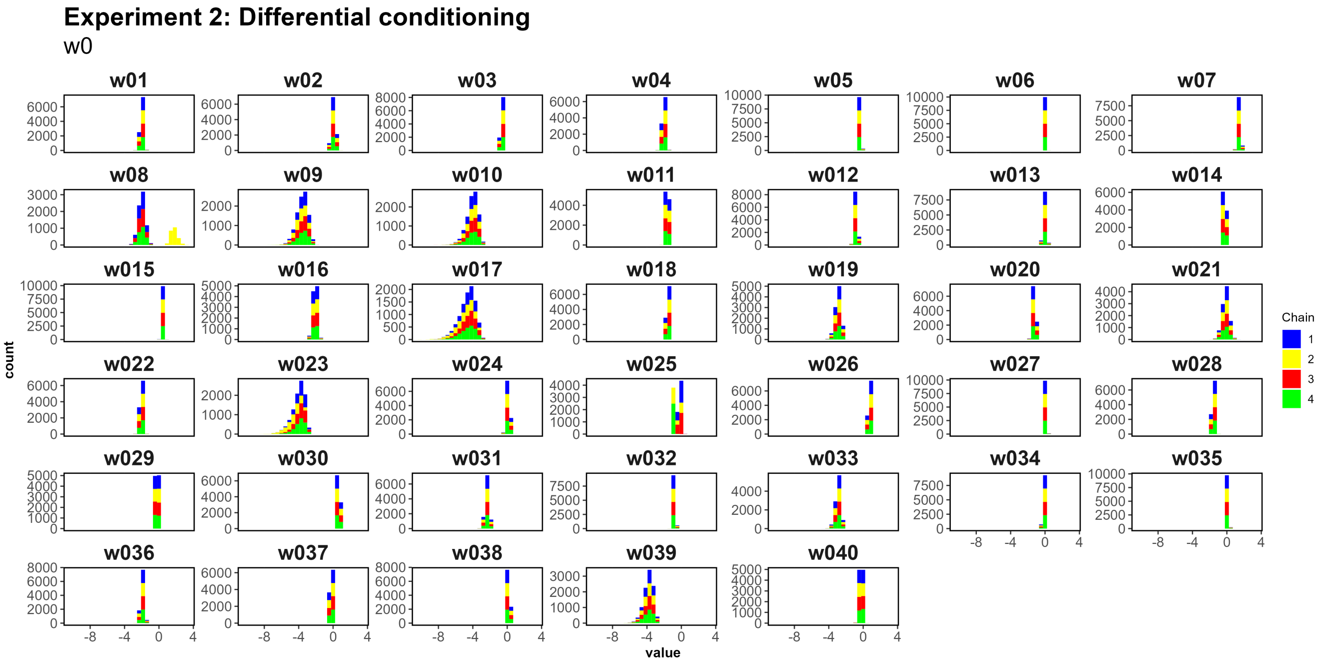

Supplementary Figure 34. The posterior distributions for person-specific learning rate $w0$ in Experiment 2.

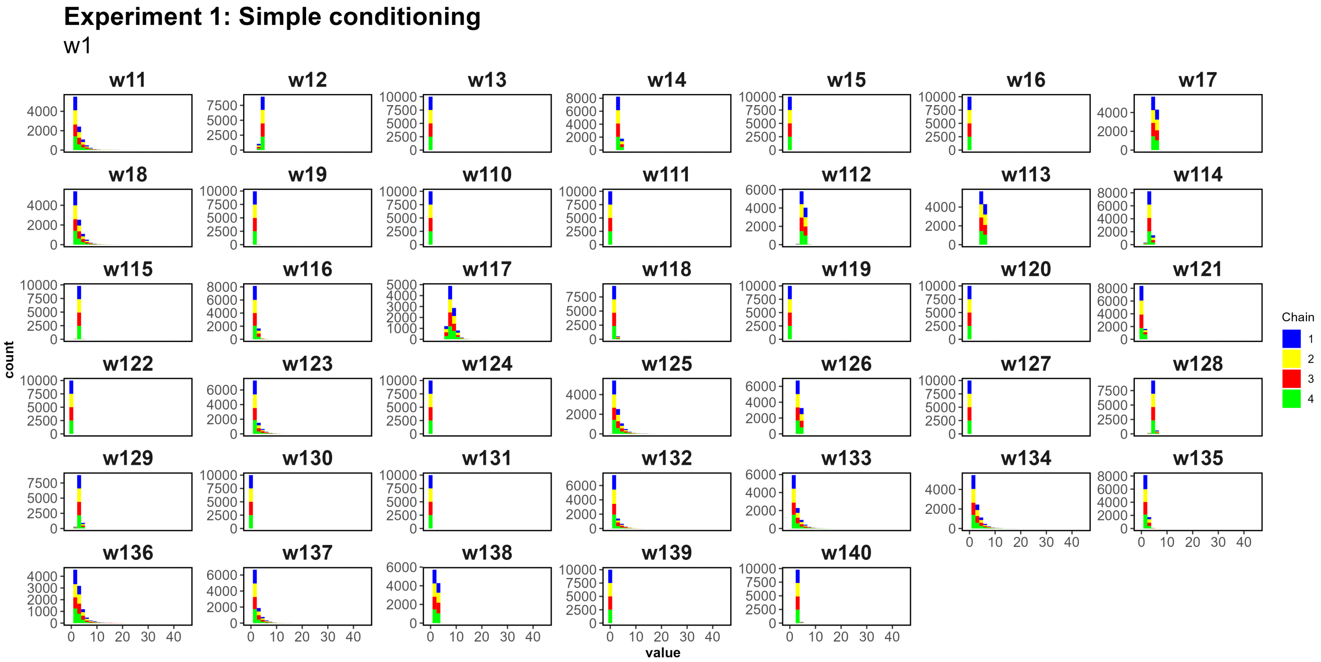

Supplementary Figure 35. The posterior distributions for person-specific learning rate $w1$ in Experiment 1.

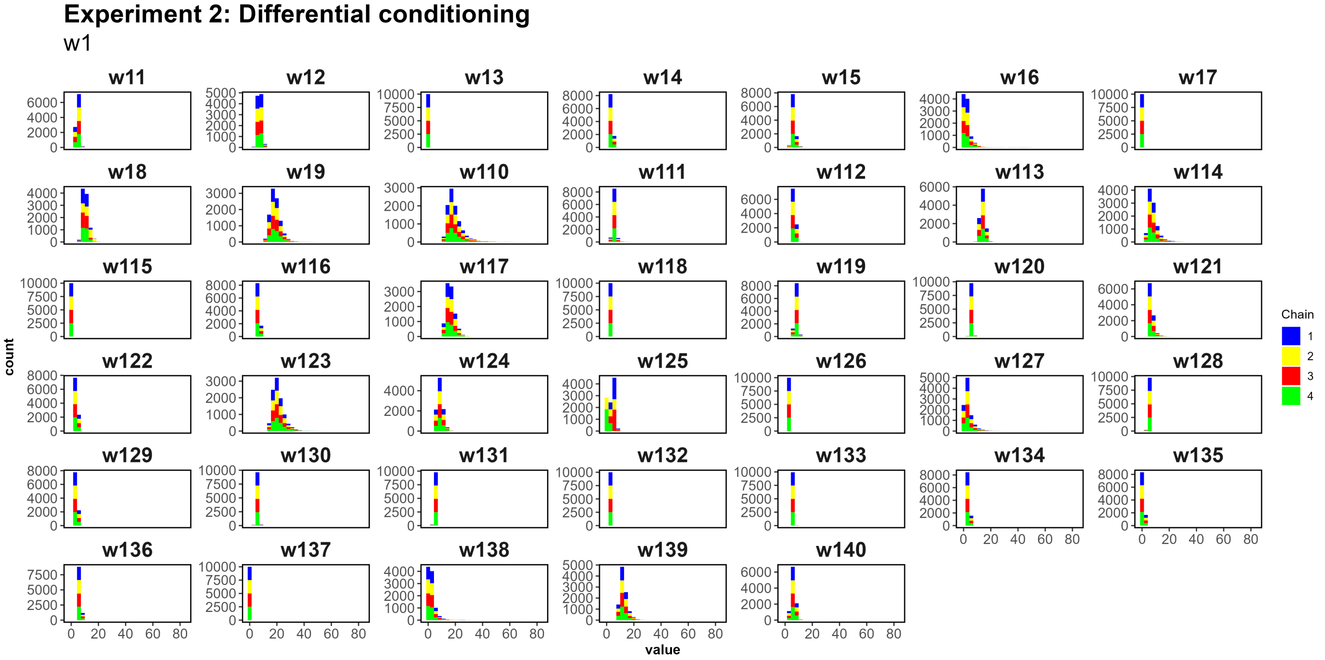

Supplementary Figure 36. The posterior distributions for person-specific learning rate $w1$ in Experiment 2.

Supplementary Note 6

Parameter recovery investigates the correspondence between the true parameters (i.e., that have been used to generate the 200 synthetic participants' data) and the estimated parameters obtained from fitting the model to the synthetic data. The four groups ($m_i = 1, \ldots, 4$) are analogous to four sub-models with different data-generating processes. By examining the result of parameter recovery, it is possible to determine whether the parameters of interest (i.e., the person-specific generalization rate λ_i and learning rate α_i) simulated by distinct sub-models are identifiable by the mixed model. Figure 37 shows that the mixed model can greatly recover λ_i simulated by all of the sub-models; nevertheless, it was unable to recover some of the α_i values simulated by the model of *Overgeneralizers*. The cause of the recovering problem for some α_i is due to the label-switching problem [a] between *Non-Learners* and *Overgeneralizers*. When λ_i is exceedingly small, all responses are shrunk to the same point, regardless of the α_i value, making λ_i and α_i indistinguishable. This implies that participants with an exceptionally flat response gradient will always be classified as *Non-Learners* because there is no indication of learning from the data.

[a] During MCMC sampling, label switching is a common identifiability issue with mixture models. Because the labels attached to the components are arbitrary, any permutation is allowed. The problem is particularly a problem if the components are hard to distinguish a priori (e.g., a normal mixture with two components and free means and variances). However, in the current study, label switching may not be a large problem because the components *Non-Learners* and *Overgeneralizers* are very strictly defined. If the problem nevertheless occurs for some participants, they will be excluded from the final analysis.

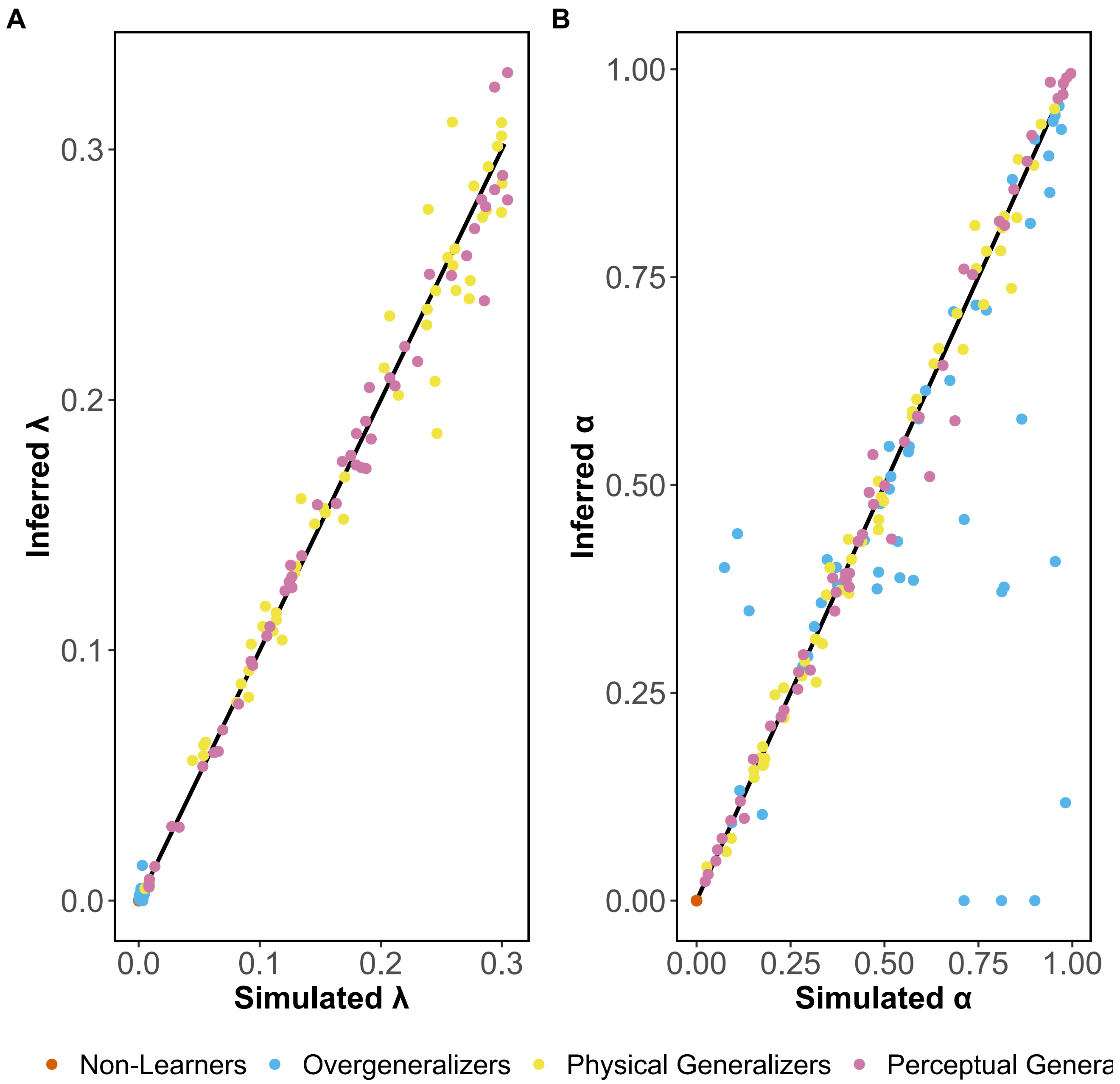

Supplementary Figure 37. The recovery plots for the generalization rate parameter λ_i (panel A) and learning rate α_i (panel B).

Supplementary Note 7

As illustrated in the manuscript, we simulated 50 participants for each latent group to investigate how well the model can identify different behavior patterns. In Figure 38, we can see that the model can very well identify the correct latent groups in most posterior samples. The problem occurs in some scenarios. First, the model will identify individuals as *Non-Learners* when the response gradient is extremely flat even though the flatness is actually caused by extremely strong generalization tendency. Second, the model will have problems disentangling *Overgeneralizers* and *Physical Generalizers* when the generalization rate is very closed to the .0052 boundary value.

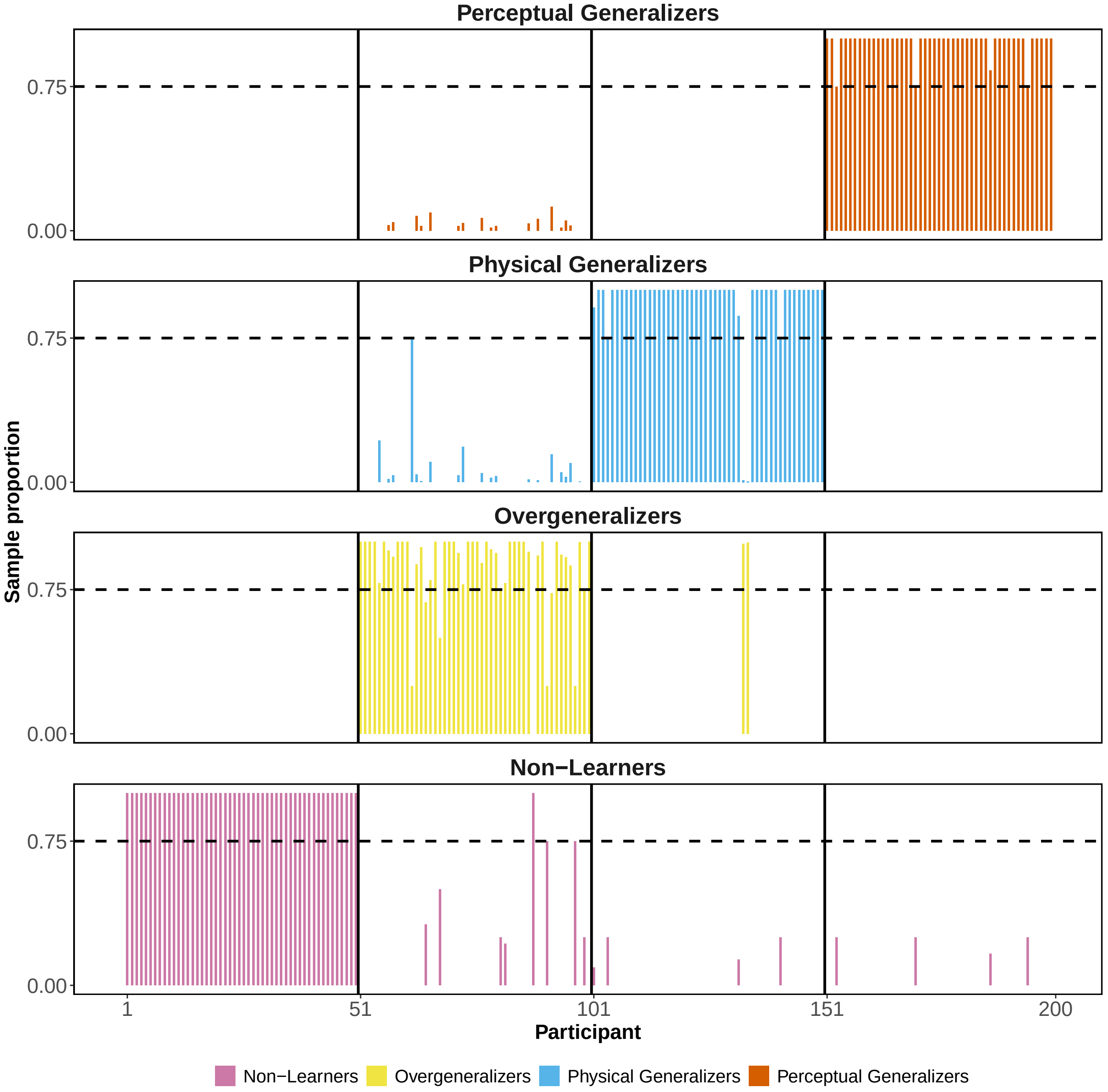

Supplementary Figure 38. Posterior samples for the group allocation parameter. Participants are deemed to have effectively allocated to one group when greater than 75% (the black dashed line) of posterior samples are observed in the same group.

Supplementary References

1. Gelman, A. & Rubin, D. B. Inference from Iterative Simulation Using Multiple Sequences. *Stat. Sci.* **7**, DOI: 10.1214/ss/1177011136 (1992).
2. Brooks, S. P. & Gelman, A. General Methods for Monitoring Convergence of Iterative Simulations. *J. Comput. Graph. Stat.* **7**, 434–455, DOI: 10.1080/10618600.1998.10474787 (1998).
