# Supplementary material for: Humans display interindividual differences in the latent mechanisms underlying fear generalization behaviour
Source: Commun Psychol. 2023 Aug 1;1:5. doi: 10.1038/s44271-023-00005-0 (PMC11290606; doi:10.1038/s44271-023-00005-0)
Supplement: Supplementary file 3 — Reporting Summary [file 44271_2023_5_MOESM3_ESM.pdf]

nature portfolio

Corresponding author(s): Kenny Yu
Last updated by author(s): 2023/06/12

Reporting Summary

Nature Portfolio wishes to improve the reproducibility of the work that we publish. This form provides structure for consistency and transparency in reporting. For further information on Nature Portfolio policies, see our Editorial Policies and the Editorial Policy Checklist.

Statistics

For all statistical analyses, confirm that the following items are present in the figure legend, table legend, main text, or Methods section.

n/a	Confirmed	
☐	☒	The exact sample size (*n*) for each experimental group/condition, given as a discrete number and unit of measurement
☒	☐	A statement on whether measurements were taken from distinct samples or whether the same sample was measured repeatedly
☒	☐	The statistical test(s) used AND whether they are one- or two-sided<br>*Only common tests should be described solely by name; describe more complex techniques in the Methods section.*
☒	☐	A description of all covariates tested
☒	☐	A description of any assumptions or corrections, such as tests of normality and adjustment for multiple comparisons
☒	☐	A full description of the statistical parameters including central tendency (e.g. means) or other basic estimates (e.g. regression coefficient) AND variation (e.g. standard deviation) or associated estimates of uncertainty (e.g. confidence intervals)
☒	☐	For null hypothesis testing, the test statistic (e.g. *F*, *t*, *r*) with confidence intervals, effect sizes, degrees of freedom and *P* value noted<br>*Give P values as exact values whenever suitable.*
☐	☒	For Bayesian analysis, information on the choice of priors and Markov chain Monte Carlo settings
☐	☒	For hierarchical and complex designs, identification of the appropriate level for tests and full reporting of outcomes
☒	☐	Estimates of effect sizes (e.g. Cohen's *d*, Pearson's *r*), indicating how they were calculated

Our web collection on statistics for biologists contains articles on many of the points above.

Software and code

Policy information about availability of computer code

Data collection	The experiment consisted of a computer task programmed in Affect 4.0.
Data analysis	R (version 4.1.1) and JAGS (version 4.3.1) were used to analyze data.

For manuscripts utilizing custom algorithms or software that are central to the research but not yet described in published literature, software must be made available to editors and reviewers. We strongly encourage code deposition in a community repository (e.g. GitHub). See the Nature Portfolio guidelines for submitting code & software for further information.

Data

Policy information about availability of data

All manuscripts must include a data availability statement. This statement should provide the following information, where applicable:

- Accession codes, unique identifiers, or web links for publicly available datasets
- A description of any restrictions on data availability
- For clinical datasets or third party data, please ensure that the statement adheres to our policy

The code for the computational model and analysis, as well as supplementary materials with additional information about the model and results, can be found at the same repository as the data: https://osf.io/sxjak/.

Human research participants

Policy information about studies involving human research participants and Sex and Gender in Research.

Reporting on sex and gender	Participants were instructed in English. They were asked to report their gender. This study included approximately equal numbers of men and women in both experiments. While we did not collect specific data on gender identity or expression, the study did not include sex- or gender-based analysis.
Population characteristics	The participants in this study were mainly undergraduate students at KU Leuven. As a result, the mean ages of the participants in the two experiments were 21.8 and 23.5, respectively. In this study, we only recruited healthy participants.
Recruitment	We recruited participants for this study through the participant recruitment system at KU Leuven as well as through posters.
Ethics oversight	SMEC

Note that full information on the approval of the study protocol must also be provided in the manuscript.

Field-specific reporting

Please select the one below that is the best fit for your research. If you are not sure, read the appropriate sections before making your selection.

☐ Life sciences ☒ Behavioural & social sciences ☐ Ecological, evolutionary & environmental sciences

For a reference copy of the document with all sections, see nature.com/documents/nr-reporting-summary-flat.pdf

Behavioural & social sciences study design

All studies must disclose on these points even when the disclosure is negative.

Study description	The study involve two laboratory experiments with typical fear conditioning paradigms. Only quantitative data (i.e., self-report fear, stimulus size estimation, and startle eyeblink response) was collected. Both the raw data and processed data for modelling and visulization can be accessed through the OSF repository: https://osf.io/sxjak/.
Research sample	The sample for this study was primarily composed of undergraduate students at KU Leuven (Experiment 1: mean age = 21.8 years, SD = 5.3, with 26 females (65%); Experiment 2: mean age = 23.5 years, SD =8.9, with 26 females (60%)).
Sampling strategy	All participants were recruited via the KU Leuven's Sona Systems and received either course credits or a monetary compensation (12 euro for Experiment 1 and 16 euro for Experiment 2).
Data collection	The data was collected and recorded using a computer. During data collection, there was always only one participant and one researcher present in the laboratory. The researcher was aware of the experimental condition and study hypothesis while the data was being collected.
Timing	The data collection was done between November 2017 and February 2018.
Data exclusions	Initially, data from three participants was excluded from the entire data analysis process due to incomplete tasks. After fitting the data to the model, our computational model divided the participants into four latent groups based on the posterior values of the group allocation parameters. Additionally, some participants whose parameters had convergence issues were also excluded from the analysis. All of these information are specifically mentioned in the manuscript or supplementary materials.
Non-participation	No participants dropped out/declined participation.
Randomization	In Experiment 1, there was only one group. In Experiment 2, participants were randomly assigned to one of two groups. In the first group, the smallest stimulus served as the CS+ and the largest stimulus served as the CS-, while the opposite was true for the second group.

Reporting for specific materials, systems and methods

We require information from authors about some types of materials, experimental systems and methods used in many studies. Here, indicate whether each material, system or method listed is relevant to your study. If you are not sure if a list item applies to your research, read the appropriate section before selecting a response.

Materials & experimental systems

n/a	Involved in the study
☒	☐ Antibodies
☒	☐ Eukaryotic cell lines
☒	☐ Palaeontology and archaeology
☒	☐ Animals and other organisms
☒	☐ Clinical data
☒	☐ Dual use research of concern

Methods

n/a	Involved in the study
☒	☐ ChIP-seq
☒	☐ Flow cytometry
☒	☐ MRI-based neuroimaging
